# C–C bond cleavage and carbonylation enabled by an NNN-pincer uranium scaffold *via* metal–arene interaction†

**DOI:** 10.1039/d5sc04248h

**Published:** 2025-07-23

**Authors:** Yue Pang, Thayalan Rajeshkumar, Rosario Scopelliti, Laurent Maron, Marinella Mazzanti

**Affiliations:** a Group of Coordination Chemistry, Institut des Sciences et Ingénierie Chimiques, École Polytechnique Fédérale de Lausanne (EPFL) CH-1015 Lausanne Switzerland marinella.mazzanti@epfl.ch; b Laboratoire de Physique et Chimie des Nano-objets, Institut National des Sciences Appliquées 31077 Toulouse Cedex 4 France laurent.maron@irsamc.ups-tlse.fr; c X-Ray Diffraction and Surface Analytics Platform, Institut des Sciences et Ingénierie Chimiques, École Polytechnique Fédérale de Lausanne (EPFL) CH-1015 Lausanne Switzerland

## Abstract

Metal–arene complexes have recently attracted an increasing interest in f-element chemistry, but the functionalization of arenes mediated by uranium–arene interactions is limited to a single example. Here, we report a new uranium–biphenylene complex supported by a bulky rigid trianionic NNN-pincer ligand in which the uranium–arene interaction is able to promote C–C bond cleavage and functionalization with CO under mild conditions to yield a U-bound 9-fluorenone. Reduction of the U(iv)-pincer complex [NNN-U(THF)Cl_2_K(THF)_3_]_2_ (1) with KC_8_, in the presence of biphenylene, results in the terminal arene complex [NNN-U(THF)(biphenylene)][K(THF)_5_] (3). DFT studies of 3 indicate the presence of two unpaired electrons located at the uranium center, in line with a U(iv) and a biphenylene dianion. Complex 3 undergoes C_aryl_–C_aryl_ bond cleavage of the biphenylene ligand, affording [NNN-U(THF)(2,2′-biphenyl)][K(THF)_2_] (4). DFT studies indicated that, due to the interaction between the biphenylene dianion and the uranium, a concerted ring opening reaction can occur on the strained four members ring to yield 4 while the uranium center retains a +IV oxidation state. Complex 4 undergoes facile CO insertion into the U–C_aryl_ bond, followed by the C_aryl_–C_carbonyl_ bond formation, yielding [NNN-U(THF)_2_(fluorenone)][K(THF)_4_] (5). This work demonstrates the potential of uranium–arene interactions to promote arene activation and functionalization.

## Abstract 2

Enabled by a bulky rigid trianionic NNN-pincer ligand, the uranium–arene interaction results in the facile cleavage of the biphenylene C–C bond and the functionalization with CO under mild conditions to yield a U-bound 9-fluorenone.

## Graphical abstract

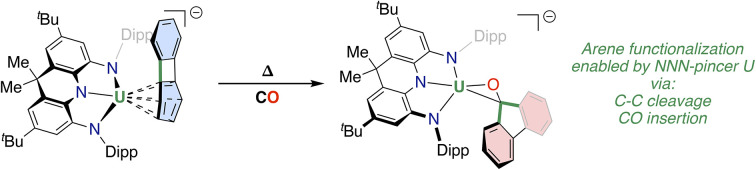

## Introduction

Metal–arene complexes have recently attracted an increasing interest in f-element chemistry.^1–13^

**Scheme 1:**
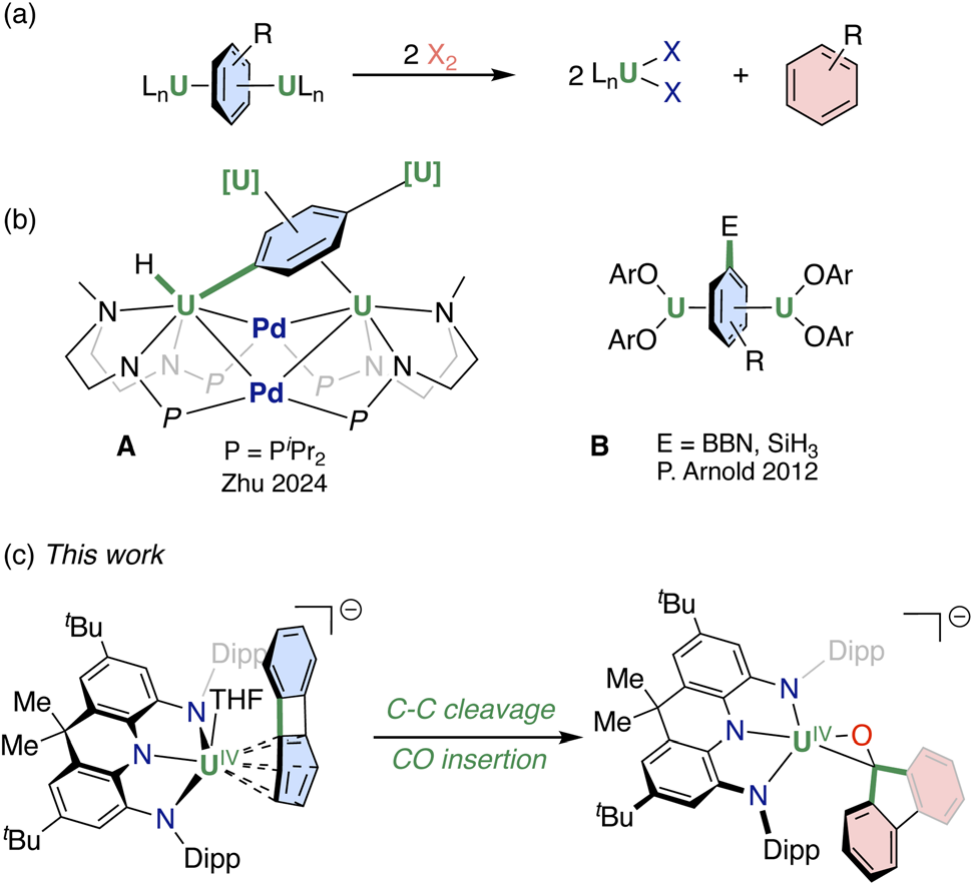
(a) Redox reactivities of uranium–arene inverse sandwich complexes; (b) examples of arene activation and functionalization enabled by uranium; (c) this work: C–C bond cleavage and carbonylation of biphenylene. Dipp = 2,6-diisopropylphenyl. The arene^*n*−^ and neutral arene are drawn in blue and red, respectively; bond cleavage and formation are drawn in bold green.

Notably, uranium–arene interactions have provided an important tool to investigate bonding modes,^9,14–21^ to stabilize very low oxidation states [U(ii) and U(i)]^12,22–26^ and rare bonding motifs.^27^ Following the seminal discovery by Diaconescu, Cummins *et al.*^28^ of uranium inverse sandwich complexes featuring a U–arene–U core supported by δ-bonding interactions, several complexes were identified, presenting bridging arene moieties with different degrees of reduction.^2,20,29–31^ The electrons stored in the arene moieties become accessible to different substrates in redox reactions, with concomitant release of the bridging arenes in the neutral form (Scheme 1a).^28,30,32,33^ In a few instances uranium–arene δ-bonding interactions were shown to lead to attractive magnetic properties^31^ and to enable rare catalytic activity.^34–36^ More recently, a unique case (A, Scheme 1b) of C–H bond oxidative addition of benzene was also reported for a heterometallic U–Pd cluster.^37^ In contrast, there is only one previous example where uranium–arene interactions have resulted in arene functionalization in uranium inverse sandwich complexes (B, Scheme 1b).^38^

Terminal actinide arenides^9,19,39,40^ are attractive candidates for promoting arene functionalization because they demonstrated relevant higher reactivity compared to bridging arenides^4^ but their chemistry remains poorly explored. We reasoned that bulky rigid pincer ligands^41^ should prevent the formation of inverse-sandwich complexes and therefore provide ideal ancillary scaffolds to isolate highly reactive terminally bound uranium arenides. We therefore set out to investigate the ability of the bulky triamide dihydroacridine-derived ligand (NNN^3−^),^42,43^ which has not yet been used in uranium chemistry, to enable access to low oxidation states and to uranium–arene interactions.

Here, we report a new terminal uranium–biphenylene complex supported by the bulky rigid trianionic NNN-pincer ligand^42,43^ in which the uranium–arene interaction is able to promote C–C bond cleavage and enable functionalization of the biphenylene moiety with CO under mild conditions (Scheme 1c).

## Result and discussion

### Uranium pincer complexes

**Scheme 2:**
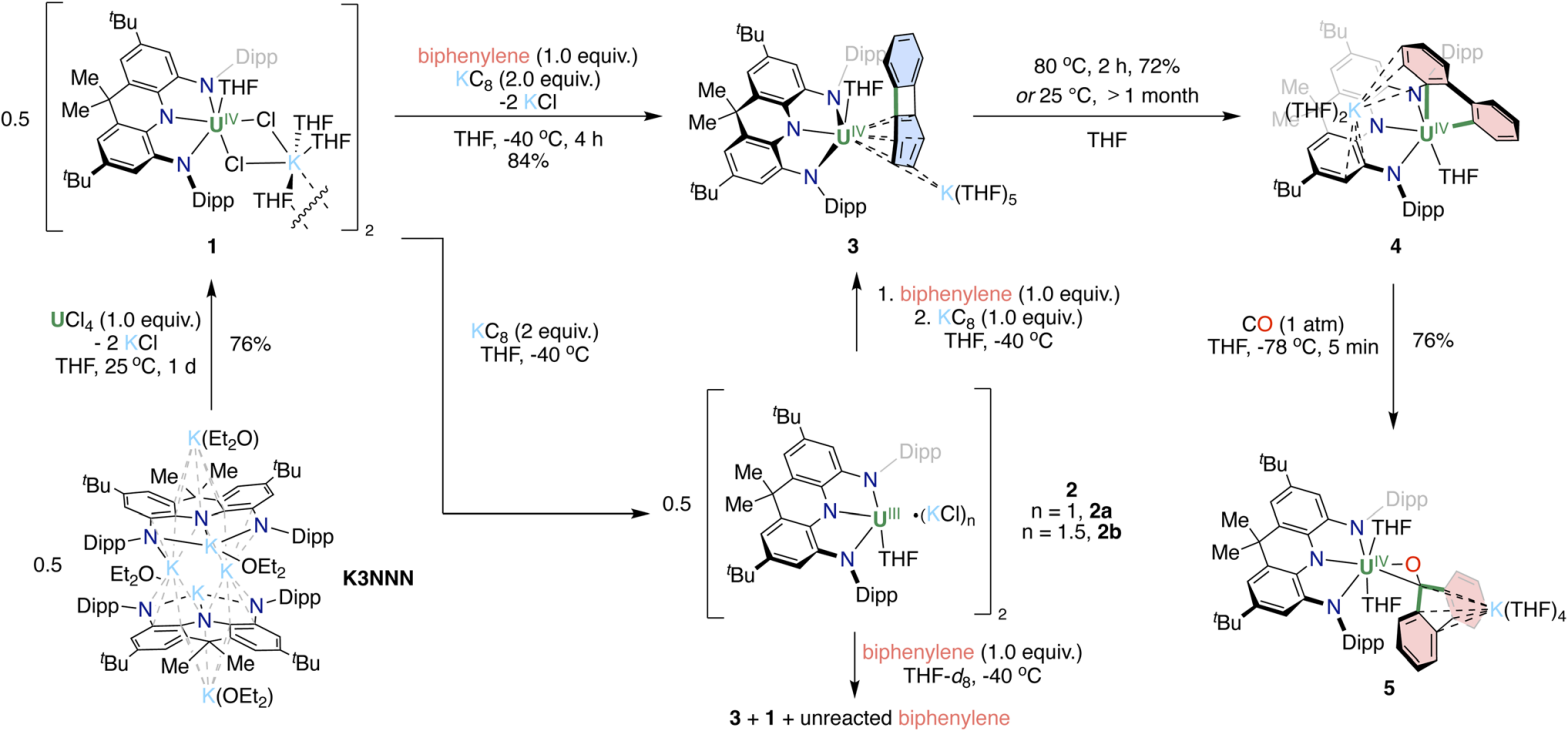
Synthesis of uranium complexes 1–5 supported by the NNN-pincer scaffold.

**Fig. 1:**
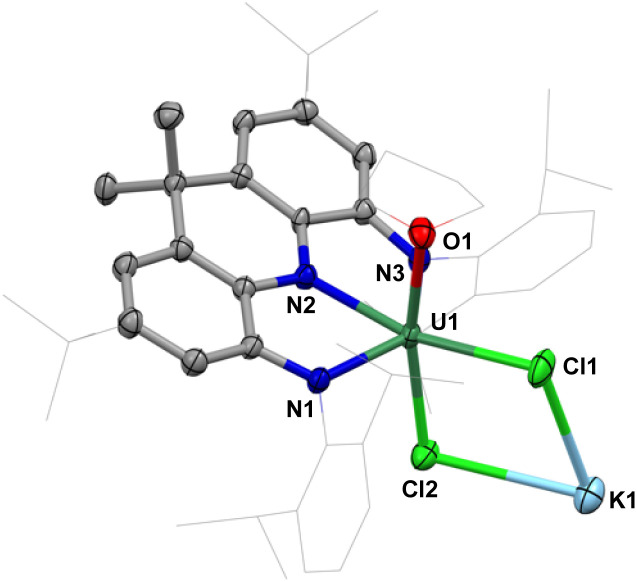
Molecular structure of the fragment [NNN-U(THF)(μ-Cl)_2_K] in 1; with thermal ellipsoids drawn at the 50% probability level. Hydrogen atoms and potassium-bound solvent molecules are omitted for clarity; ^*t*^Bu and Dipp groups of the pincer backbones and uranium-bound THF molecules are drawn in a wireframe style (the figures below follow the same style).

The reaction of the potassium salt of the NNN pincer ligand (K_3_NNN) with UCl_4_ in a 1 : 1 ratio in THF at room temperature yielded the U(iv) complex 1 that was isolated as a green solid in 76% yield from a Et_2_O/hexane mixture (Scheme 2). Single crystals of 1 suitable for XRD analysis were obtained from a mixture of THF and *n*-hexane at −40 °C. X-ray studies revealed that 1 has a dimeric structure in the solid state, where two monomeric [NNN-U(THF)(μ-Cl)_2_K(THF)_3_] units are connected by two bridging THF molecules from two different moieties (Fig. 1). In complex 1, each uranium center is hexa-coordinated by the three N atoms of the pincer ligand, one oxygen of THF and two chlorides bridging the uranium and the potassium cation. The U–N bond distances range from 2.266(3) to 2.304(3) Å and are similar to those reported for the U(iv) complex of NON (NON = [4,5-bis(2,6-diisopropylanilino)-2,7-di-*tert*-butyl-9,9-dimethylxanthene]) rigid pincer system (2.297(4) and 2.306(4) Å).^44^

With complex 1 in hand, we explored its reduction to investigate if the NNN pincer scaffold could support uranium in lower oxidation states. ^1^H NMR spectroscopic studies showed that when 1 was treated with 2 equiv. of KC_8_ in THF-*d*_8_ at −40 °C, full consumption of 1 was reached in 2 h, resulting in the formation of new species (Fig. S5†).

**Fig. 2:**
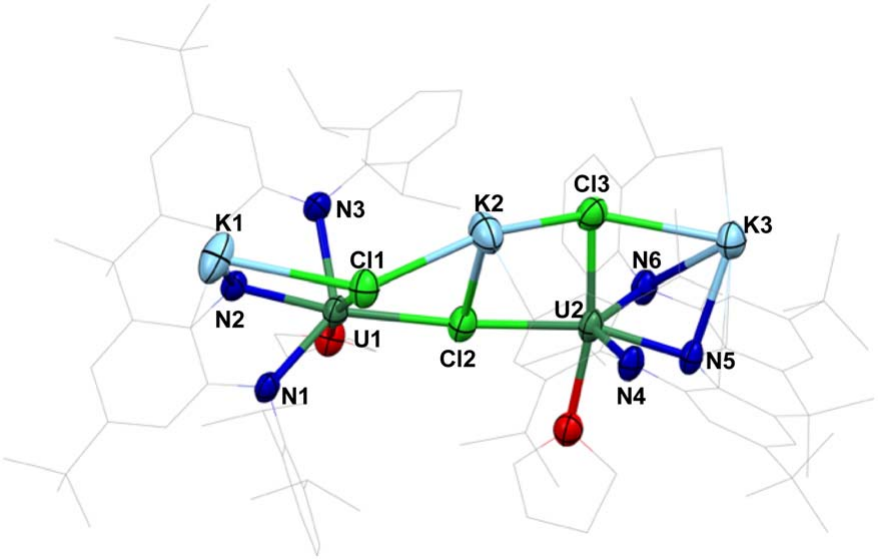
Molecular structure of [NNN-U^III^(THF)]_2_(KCl)_3_(THF)_2_(Et_2_O)_2_, 2b; with thermal ellipsoids drawn at the 50% probability level.

A mixture of dark purple and brown crystals was obtained upon addition of *n*-hexane to the concentrated THF filtrate of the reaction mixture at −40 °C. XRD analysis of a dark brown single crystal showed the presence of a dimeric U(iii) species, [NNN-U^III^Cl(THF)K(THF)_3_]_2_ (2a), consisting of two [NNN-U^III^(THF)] units bridged by two KCl molecules (Fig. S26†), however, the poor quality of the single crystal prevents further discussion of the structure of 2a. Furthermore, dark purple single crystals of [NNN-U^III^(THF)]_2_(KCl)_3_(THF)_2_(Et_2_O)_2_ (2b) suitable for XRD analysis were obtained from a concentrated Et_2_O solution of the isolated material at −40 °C, showing the presence of a dimeric U(iii) species bridged by three KCl molecules (Fig. 2). The U–N bond lengths in 2b (2.385(10) to 2.417(9) Å) are significantly longer than those in 1 (2.266(3) to 2.304(3) Å), in accordance with the change of oxidation states from +IV (1) to +III (2b). Overall, the presence of variable numbers of bridging KCl molecules complicates the isolation and characterization of the U(iii) species [NNN-U^III^(THF)]_2_(KCl)_*n*_ (2) obtained from the reduction of 1. ^1^H NMR spectroscopic studies of THF-*d*_8_ solutions of complex 2 showed that the complex decomposes slowly at −40 °C, but rapidly at room temperature.

We also attempted the direct synthesis of the U(iii) pincer complex from UI_3_(THF)_4_ (Scheme S2†). The reaction of K3NNN with UI_3_(THF)_4_ is very slow and did not reach complete conversion after 2 weeks (Fig. S11†). Dark brown crystals were obtained from the reaction mixture, showing the presence of NNN-U^III^(THF)_3_ (2c) (Fig. S27†). Complex 2c decomposes rapidly at room temperature rendering difficult to increase the rate of its formation.

### Uranium-terminal biphenylene complex

With the U(iii) pincer complex in hand we set out to isolate a terminal arenide complex and pursued the reactivity of the U(iii) species 2 with biphenylene. Notably, metalla–biphenylene complexes provide an attractive target for C–C bond activation and functionalization, due to the weak character of the bond, which has resulted in a rich chemistry of transition-metal promoted C–C bond cleavage and functionalization,^45,46^ including a very recent example with rare-earth complexes^47^ but has not been investigated in actinide chemistry.

The addition of 1 equiv. biphenylene to a THF-*d*_8_ solution of the *in situ* generated U(iii) species 2 at −40 °C resulted in an immediate color change from dark purple to dark brown. The ^1^H NMR spectrum of the reaction mixture showed the resonances assigned to the complex [NNN-U(THF)(biphenylene)][K(THF)_5_] (3), together with signals assigned to the U(iv) precursor 1 in a ratio of 1 : 1 and to signals of unreacted biphenylene (Scheme 2 and Fig. S10†). The addition of KC_8_ (1 equiv.) to the reaction mixture led to the full conversion of 1 and biphenylene into complex 3, as indicated by ^1^H NMR spectroscopy (Scheme 2 and Fig. S8†). Dark brown needles of the complex [NNN-U(THF)(biphenylene)][K(THF)_5_] (3) were isolated in 76% yield on a preparative scale from the THF filtrate upon addition of *n*-hexane at −40 °C.

The formation of the terminally U-bound arenide 3 and of the U(iv) complex 1 from the reaction of the U(iii) species 2 with biphenylene is the result of the two-electron reduction of biphenylene by two U(iii) centers (Scheme S1†). The observed reactivity differs from previously reported reactions of U(iii) complexes with arenes leading to inverse sandwich complexes of uranium involving different degree of arene reduction ranging from arene^2−^ to arene^4−^.^2,31,48^ It is likely that, in the reaction of the U(iii) species 2 with biphenylene, the bulky NNN ligand prevents the formation of an inverse sandwich complex and leads to a terminal [U(iv)-biphenylene^1−^] species that is rapidly reduced by a second uranium(iii) complex to afford the final U(iv)-biphenylene^2−^. It is worth noting that a few terminally bound arenides were previously reported for uranium.^16,19^

Complex 3 can also be obtained in a similar yield by adding directly 2 equiv of KC_8_ to a THF solution of complex 1 and 1 equiv. biphenylene at −40 °C (Scheme 2).

**Fig. 3:**
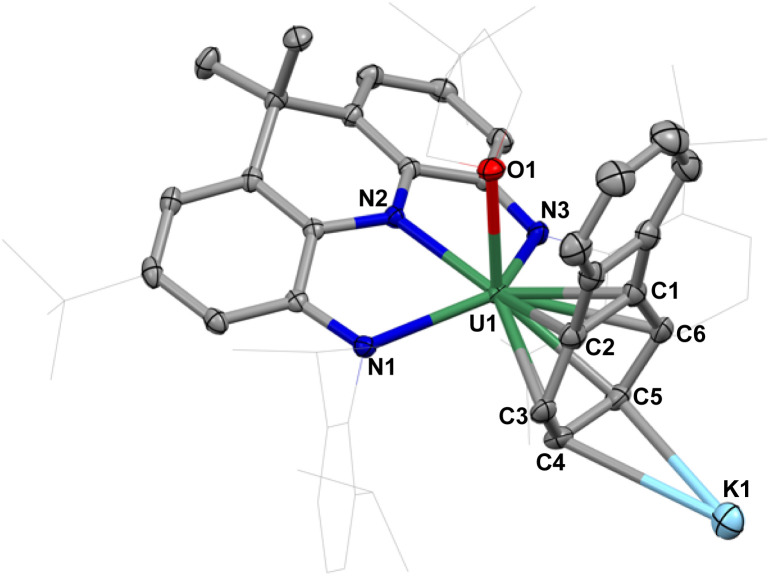
Molecular structure of [NNN-U(THF)(biphenylene)][K(THF)_5_], 3; with thermal ellipsoids drawn at the 50% probability level.

The solid-state molecular structure of 3 (Fig. 3) revealed the presence of a heterobimetallic complex where the biphenylene ligand binds a uranium center and a THF-solvated potassium cation. In complex 3, the uranium center is bound by three N atoms of the NNN ligand, one oxygen of THF and the biphenylene ligand in a η^6^ fashion, with U–C_aryl_ bond distances ranging from 2.628(2) to 2.8089(19) Å. The solvated K^+^ is interacting with the C4 and C5 atoms of the coordinated phenyl ring. As a result, the coordinated phenyl ring is slightly folded towards the U center with an angle of 14.38° along C3–C6 axis. The bond lengths of C1–C2 (1.531(3) Å) and C4–C5 (1.461(3) Å) in the coordinated ring are noticeably longer than those in the uncoordinated ring (1.436(3) and 1.394(4) Å). These metrical parameters are comparable to those found in the previously reported heterometallic cluster with a [U(iv)–Pd(0)]_2_[biphenylene] core,^37^ suggesting the presence of a doubly reduced biphenylene with the uranium center in the +IV oxidation state. The U–N bond lengths in 3 (2.3355(15) to 2.3963(16) Å) are significantly longer than those in 1 (2.266(3) to 2.304(3) Å), but still in the range of U(iv)–N_amido_ distances reported for the U(iv) complexes supported by a sterically congested PNP scaffold (PNP = bis[2-(diisopropylphosphino)-4-methylphenyl]amido ligand) (2.343(7)-2.411(3) Å).^49^ The lengthening of the U–N bond distances in 3 compared to 1 is probably due to the increased steric constraints imposed by the coordination of the biphenylene ligand rather than to the presence of a reduced uranium center. By sharp comparison to the flat pincer ligand in 1 (5.04°), the backbone in 3 is noticeably bent with an angle of 32.87°, thus minimizing the steric repulsion between two Dipp side arms and the coordinated biphenylene. This further suggests that the U–arene terminal binding mode in 3 is due to the bulky nature of the supporting NNN ligand.

### C–C bond cleavage and carbonylation of the U-bound biphenylene

Considering the high reactivity of arenide complexes of uranium towards different substrates,^2,19,28,32,33,38,50,51^ we investigated the stability and reactivity of 3.

Although 3 remained stable in a THF-*d*_8_ solution at −40 °C for at least one week, it evolved slowly into a new species at room temperature, as monitored by ^1^H NMR spectroscopy (Fig. S15†). When a THF-*d*_8_ solution of 3 was heated at 80 °C, the complete conversion of 3 was achieved in 2 h, and the solution turned from dark brown to orange (Scheme 2 and Fig. S16†).

**Fig. 4:**
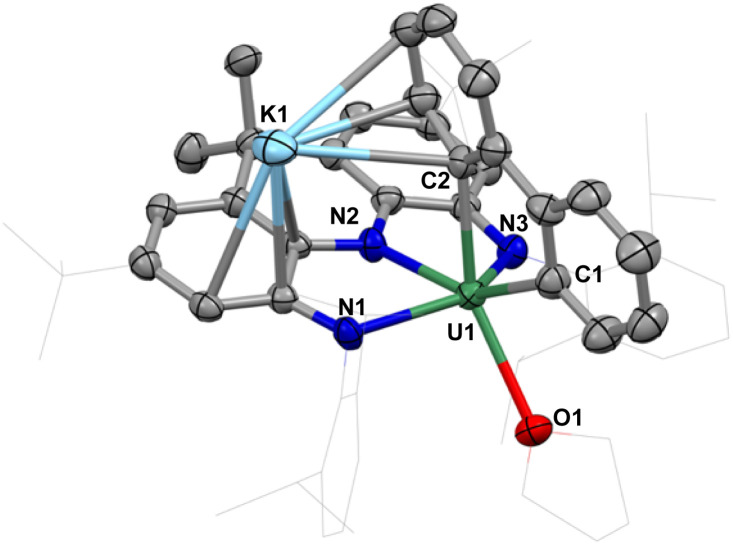
Molecular structure of [NNN-U(THF)(2,2′-biphenyl)][K(THF)_2_], 4; with thermal ellipsoids drawn at the 50% probability level.

The complex [NNN-U(THF)(2,2′-biphenyl)][K(THF)_2_] (4) was isolated as a brick-red microcrystalline solid on a preparative scale from a mixture of THF and *n*-hexane at −40 °C in 72% yield. As revealed by the X-ray determined solid-state molecular structure of 4, the coordinated biphenylene in 3 undergoes C–C bond cleavage, affording the 9-uranafluorene derivative (Fig. 4). The resulting biphenyl ligand sits in a pocket between the two aryl substituents, to reduce steric hindrance. The U–C_aryl_ bond distances of 2.491(5) and 2.520(5) Å fall in the range of previously reported U(iv)–aryl and U(iv)–benzyne complexes (2.340(5) to 2.650(7) Å).^52–55^ The U–N bond distances in 4 (2.322(4) to 2.342(3) Å) are shorter than those found in complex 3 (2.3355(15) to 2.3963(16) Å), but longer than those in complex 1 (2.266(3) to 2.304(3) Å), manifesting the moderate steric hindrance of 2,2′-biphenyl in the coordination sphere of the uranium center. The solvated K^+^ binds to one of the phenyl rings of the pincer backbone and to one of the phenyl rings in the biphenyl in η^3^ fashion.

Actinide aryl complexes remain rare^52–58^ and most previously reported actinide aryl species display rather low thermal stability, due to the ionic nature of the An–C bonds. On the contrary, no decomposition was observed for complex 4 in THF-*d*_8_ at room temperature over one week. The observed remarkable stability of the U–C bonds in 4 is probably the result of the chelating nature and steric shielding of the pincer scaffold.

The formation of complex 4 resembles an oxidative addition of the C–C bond of biphenylene to the metal center, but computational studies (see below) indicate that the cleavage of the C–C bond involves a concerted mechanism where the oxidation state of uranium remains unchanged. The C–C cleavage of biphenylene has not yet been reported for an actinide complex^37^ but a relevant example of distal C–C cleavage has been recently reported by Hayton and coworkers for U(iv)- and Th(iv)-cyclopropyl complexes.^59,60^

Since the insertion of small molecules into the highly reactive U–C bonds, such as CO, CO_2_ and carbodiimides, is documented,^56,61–65^ we decided to investigate the possibility of functionalizing the bound 2,2′-biphenyl ligand.

**Fig. 5:**
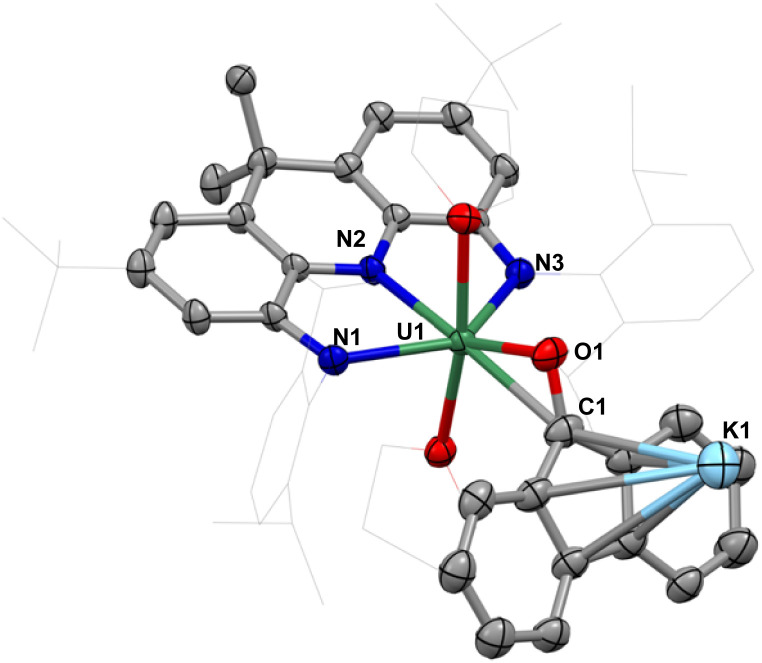
Molecular structure of [NNN-U(THF)_2_(fluorenone)][K(THF)_4_], 5; with thermal ellipsoids drawn at the 50% probability level.

A THF-*d*_8_ solution of 4 was exposed to 1 atm atmosphere of CO at −78 °C, resulting in an immediate color change from orange to dark red (Scheme 2). The ^1^H NMR spectrum of the reaction mixture showed the disappearance of the signals assigned to 4 and concomitant formation of a new species (Fig. S20†). The complex [NNN-U(THF)_2_(fluorenone)][K(THF)_4_] (5) was isolated as a dark red microcrystalline solid on a preparative scale from a mixture of THF and *n*-hexane at −40 °C in 76% yield. The solid-state molecular structure of 5 revealed the presence of an heterobimetallic complex with a 9-fluorenone ligand binding the K and U(iv) cations (Fig. 5). The uranium center in 5 is coordinated by the NNN pincer ligand, the anionic carbon and oxygen atoms of the 9-fluorenone ligand, and the oxygens of two THF. The oxidation state of the uranium centre remains unchanged. The 9-fluorenone is coordinated to the uranium center in a η^2^ fashion, with the U1–O1 and U1–C1 bond lengths of 2.166(8) and 2.659(12) Å. The U1–C1 bond is significantly elongated compared to U–C_aryl_ bond distances found in 4 but still in the range of U(iv)–C bond distances found in previously reported U(iv) aryl complexes (2.340(5) to 2.650(7) Å).^53,55^ The C1–O1 bond distance of 1.392(15) Å is significantly longer compared to that found in the free 9-fluorenone (1.217(4) and 1.222(4) Å),^66^ but comparable to the values found in metal-bound formyl groups (in [K_2_{[U-(OSi(O^*t*^Bu)_3_)_3_]_2_(μ-CH_2_O)(μ-O)}] (1.31(3) Å)^67^ and [(1,2,4-(Me_3_C)_3_C_5_H_2_)_2_Ce](μ-OCH_2_) (1.39(1) Å)),^68^ suggesting the presence of a C–O single bond. Besides, the solvated K^+^ binds to the central five-membered ring in the 9-fluorenone in a η^5^ fashion.

In the transformation from 4 to 5, the insertion of CO into one of the U–C_aryl_ bonds is followed by the formation of the C_aryl_–C_carbonyl_ bond, and the resultant dianionic 9-fluorenone fragment binds to the U(iv) center. Reactions of CO with metallafluorene complexes to yield 9-fluorenone were previously reported for a few transition-metal systems of Co, Rh, and more recently, Au^69–71^ but required high CO pressure (*e.g.* 10 atm for Au^71^) and/or high temperatures (*e.g.* 120 °C for Co^70^ and Rh^69,70^).

### Computational studies

**Scheme 3:**
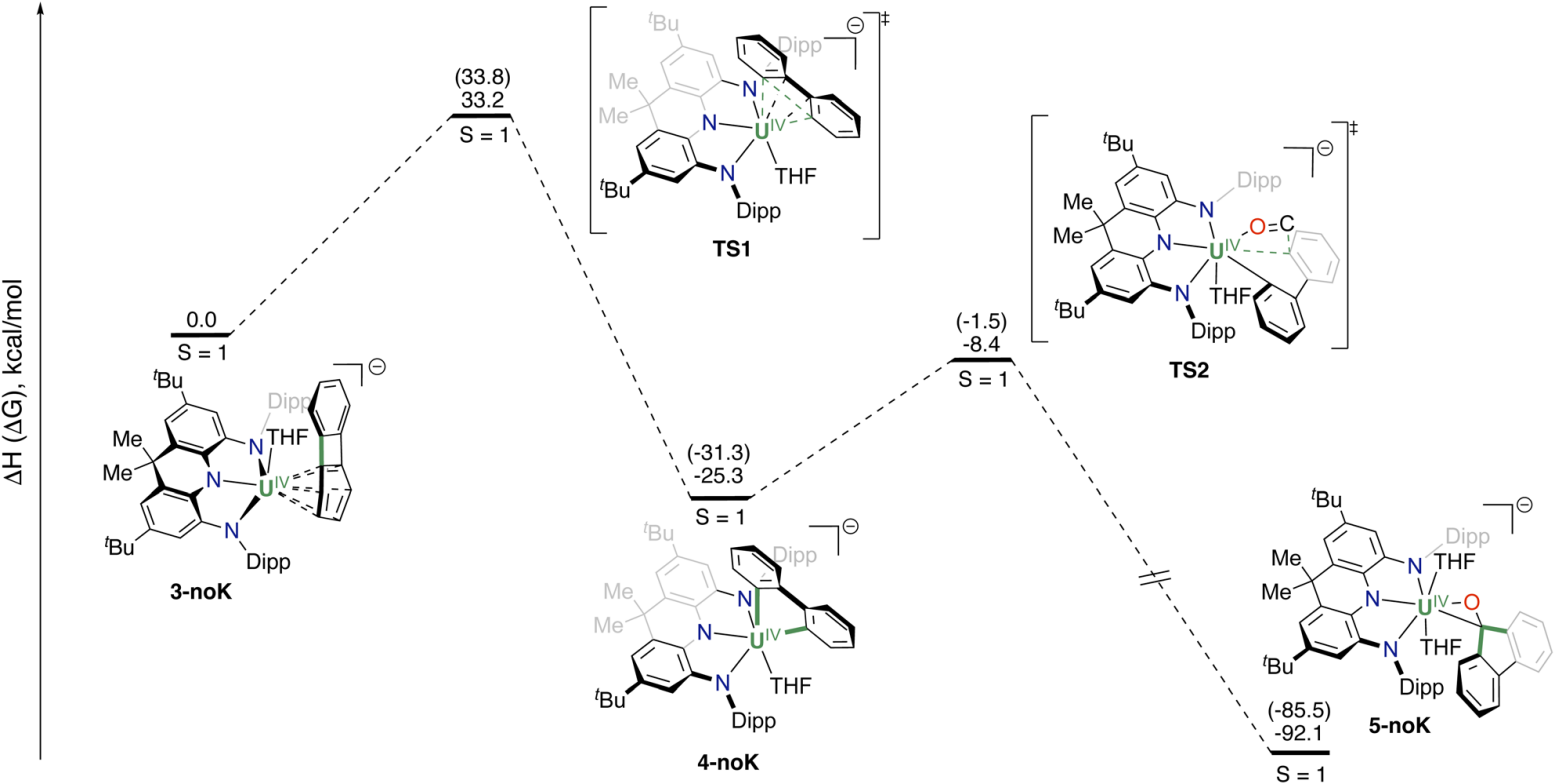
Computed enthalpy profiles (Gibbs free energy in brackets) for the formation of 4-noK and 5-noK from 3-noK.

DFT calculations (B3PW91 functional) were carried out to understand the formation of complex 3 from 1 and to gain more insights into the formation of complexes 4 and 5, from complex 3. The formation of the U(iii) analog of complex 3 (Fig. S30†), that is a U(iii) complex of the neutral biphenylene ligand, is almost athermic in Gibbs free energy (1.9 kcal mol^−1^). The subsequent reduction of this U(iii) intermediate is exergonic by 4.4 kcal mol^−1^, making the overall transformation exothermic by 2.5 kcal mol^−1^, and indicating that the formation of a U(iii) intermediate is indeed plausible. Complex 3 was optimized without the potassium counterion (3-noK) and the obtained geometry is in good agreement with the experimental one. This complex has a triplet ground state, with two unpaired electrons located at the uranium center, in line with a U(iv) and a biphenylene dianion. Due to the interaction between the biphenylene dianion and the uranium (Scheme 3), a ring opening (RO) reaction can occur on the four-membered ring, which is the most strained one *via*TS1. The associated barrier is 33.2 kcal mol^−1^ in enthalpy (33.8 kcal mol^−1^ in Gibbs free energy) in line with a slow reaction at room temperature, but faster at 80 °C as carried out experimentally. TS1 was located on the triplet PES and the two unpaired electrons were found to be located at the uranium center, in line with a U(iv) so that the RO is a concerted reaction. This can be explained by the presence of the dianionic charge of the biphenylene, where the two extra π electrons are used together with the two σ electron of the C_aryl_–C_aryl_ bond to allow this RO reaction in a concerted manner. Following the intrinsic reaction coordinate, it yields the thermodynamically stable complex 4-noK (−25.3 kcal mol^−1^ in enthalpy and −31.3 kcal mol^−1^ in Gibbs free energy), where two U–C_aryl_ bonds are formed, and the uranium remains in the +IV oxidation state. Complex 4-noK can then react with CO *via*TS2 with an associated barrier of 16.9 kcal mol^−1^ in enthalpy (29.8 kcal mol^−1^ in Gibbs free energy), in line with a facile reaction. This reaction is the CO insertion into two U–C_aryl_ bonds. At the transition state, one of the U–C_aryl_ bonds is elongated and at the same time the C_aryl_–C_carbonyl_ bond starts to be formed with the nucleophilic assistance of the oxygen interaction with the uranium center. Therefore, the carbon of CO displays a positive charge with an empty orbital. The latter can overlap with the π electron of the carbon of the second U–C_aryl_ bond. This explains why following the intrinsic reaction coordinate from TS2, the CO insertion is accompanied by a cyclization, directly yielding complex 5-noK, which is thermodynamically highly favorable (−66.8 kcal mol^−1^ in enthalpy and −54.2 kal mol^−1^ in Gibbs free Energy).

## Conclusions

In summary, we have shown that the rigid bulky triamide pincer ligand NNN^3−^ allows to access a highly reactive uranium(iii) complex 2 that readily reacts with biphenylene to yield a terminally-bound arenide complex 3. Complex 3 can also be conveniently prepared by reacting the U(iv) chloride pincer complex 1 with excess KC_8_ and biphenylene. The electronic and molecular structure of 3 indicate the presence of uranium in the +IV oxidation state and of a doubly reduced biphenylene ligand. The coordinated biphenylene in 3 undergoes rapid C–C bond cleavage affording the 9-uranafluorene derivative 4. DFT studies indicated that, due to the interaction between the biphenylene dianion and the uranium, a concerted ring opening reaction can occur on the strained four members ring that involves the two extra π electrons stored in the biphenylene to yield 4 while the uranium centre retains a +IV oxidation state. Complex 4 was found to react with CO in mild conditions *via* migratory insertion of CO into the U–C bonds to yield U-bound 9-fluorenone 5. The observed reactivity provides the first example of C–C cleavage enabled by uranium–arene interactions. These results highlight the ability of the triamide pincer ligand to stabilize highly reactive uranium species and to enable the activation of unreactive bonds without accessing hard-to-handle low oxidation states. This work also demonstrates the potential of uranium–arene interactions to promote arene activation and functionalization.

## Author contributions

Y. P. designed and carried out all the experiments and analyzed the data; M. M. designed and supervised the project; T. R. and L. M. carried out the computational study; R. S. measured and analyzed the X-ray data, Y. P. and M. M. wrote the manuscript with contributions of all authors, and all authors have given approval for the final version of the manuscript.

## Conflicts of interest

There are no conflicts to declare.

## Supplementary Material

SC-OLF-D5SC04248H-s001

SC-OLF-D5SC04248H-s002

SC-OLF-D5SC04248H-s003

## Data Availability

Synthetic details, analytical data including depictions of all spectra and coordinate data of all computationally optimised species, are documented in the ESI.† Crystallographic data is made available *via* the CCDC. The data that support the findings of this study are openly available in the Zenodo repository at DOI: https://www.doi.org/10.5281/zenodo.16447740.
