# Supplementary material for: C–C bond cleavage and carbonylation enabled by an NNN-pincer uranium scaffold via metal–arene interaction
Source: Chem Sci. 2025 Jul 23;16(35):16101–9. doi: 10.1039/d5sc04248h (PMC12330830; doi:10.1039/d5sc04248h)
Supplement: SC-016-D5SC04248H-s002 [file SC-016-D5SC04248H-s002.pdf]

Supplementary Information (SI) for Chemical Science.
This journal is © The Royal Society of Chemistry 2025

Supporting Information

C−C Bond Cleavage and Carbonylation Enabled by an NNN-Pincer Uranium Scaffold via Metal-Arene Interaction

Yue Pang,[a] Thayalan Rajeshkumar,[b] Rosario Scopelliti,[c] Laurent Maron,*[b] and Marinella Mazzanti*[a]

[a]Group of Coordination Chemistry, Institut des Sciences et Ingénierie Chimiques, École Polytechnique Fédérale de Lausanne (EPFL), CH-1015 Lausanne, Switzerland.

[b]Laboratoire de Physique et Chimie des Nano-objets, Institut National des Sciences Appliquées, 31077 Toulouse, Cedex 4, France.

[c]X-Ray Diffraction and Surface Analytics Platform, Institut des Sciences et Ingénierie Chimiques, École Polytechnique Fédérale de Lausanne (EPFL), CH-1015 Lausanne, Switzerland.

*Email to whom correspondence should be addressed: marinella.mazzanti@epfl.ch

Table of Contents

S1. General Information 3
S2. Synthesis and Reactivity Studies 4
S2.1. Synthesis of [NNN-3K(Et_2O)$_2$]$_2$ (**K3NNN**) 4
S2.2. Synthesis of [NNN-U(THF)Cl_2K(THF)$_3$]$_2$ (**1**) 4
S2.3. Synthesis of U(III) pincer complexes **2** 4
S2.4. Synthesis of [NNN-U(THF)(biphenylene)][K(THF)$_5$] (**3**) 6
S2.5. Synthesis of [NNN-U(THF)(2,2'-biphenyl)][K(THF)$_2$] (**4**) 7
S2.6. Synthesis of [NNN-U(THF)$_2$(fluorenone)][K(THF)$_4$] (**5**) 7
S3. NMR Spectroscopic Data 9
S3.1. NMR spectrum of isolated **K3NNN** 9
S3.2. NMR spectra of isolated [NNN-U(THF)Cl_2K(THF)$_3$]$_2$ (**1**) 10
S3.3. NMR spectra of the U(III) **2** 12
S3.4. NMR spectra of [NNN-U(THF)(biphenylene)][K(THF)$_5$] (**3**) 19
S3.5. NMR spectra of [NNN-U(THF)(2,2'-biphenyl)][K(THF)$_2$] (**4**) and its reaction with CO 24
S3.6. NMR spectra of isolated [NNN-U(THF)$_2$(fluorenone)][K(THF)$_4$] (**5**) 28
S4. X-ray Crystallographic Data 32
S5. EPR of complexes **2** and **3** 36
S6. Computational Details 37
S7. References 40

S1. General Information

Unless otherwise noted, all manipulations were carried out under argon atmosphere by means of Schlenk techniques and an MBraun glovebox equipped with a -40 ^{o}C freezer and a cold well. The water and oxygen levels were invariable kept below 0.1 ppm. Glassware was dried overnight at 140 °C before use.

Caution: Depleted uranium (primary isotope ^{238}U) is a weak *α*-emitter (4.197 MeV) with a half-life of 4.47 x 10^9 years. Manipulations and reactions should be carried out in monitored fume hoods or in an inert glovebox in a radiation laboratory equipped with *α*- and *β*-counting equipment.

NMR spectra were recorded on Bruker 400, 500, or 600 MHz spectrometers and referenced to residual solvent resonances of THF (THF-d_8) in Pyrex NMR tubes adapted with J-Young valves.

Elemental analyses were performed under an inert atmosphere of nitrogen with a ThermoScientific Flash 2000 Organic Elemental Analyzer.

EPR analyses were performed on a Bruker Elexsys E500 spectrometer working at 9.4 GHz frequency with an Oxford ESR900 cryostat for 4-300 K operation.

Starting materials: Unless otherwise noted, reagents were purchased from commercial suppliers and used without further purification. Anhydrous solvents were purchased from Sigma Aldrich and further distilled from potassium/benzophenone (THF, Et_2O and toluene), sodium sand/benzophenone (*n*-hexane). THF-d_8 for NMR spectroscopy was purchased from Cortecnet, distilled over potassium/benzophenone, and degassed via freeze-pump-thaw cycles. Depleted uranium turnings and UO_3 were purchased from IBILABS, Florida, USA. UCl_4[1], and KC_8[2] were prepared as previously described. Carbon monoxide (N47 Bt-S 10/200) was purchased from Carbagas. 2,7-Di-*tert*-butyl-*N*^{4},*N*^{5}-bis(2,6-diisopropylphenyl)-9,9-dimethyl-9,10-dihydroacridine-4,5-diamine (**H3NNN**) was prepared according to the literature procedure, and the work-ups were performed in a glovebox specially for aqueous reactions.[3]

X-ray crystallography data for the analyzed crystal structures were selected and mounted on various Rigaku diffractometers (XtaLAB Synergy R, DW system, HyPix-Arc 150 detector or SuperNova, Dual, Cu at home/near, AtlasS type detectors). The crystals were kept at a steady T = 140.00(10) K during data collection. Data were measured using ω scans with Cu K_α radiation. The diffraction patterns were indexed and the total number of runs and images were based on the strategy calculation from the program CrysAlisPro 1.171.43.137a (Rigaku OD, 2024)[4]. The unit cells were refined using CrysAlisPro 1.171.43.137a (Rigaku OD, 2024)[4]. Data reduction, scaling and absorption corrections were performed using CrysAlisPro 1.171.43.137a (Rigaku OD, 2024)[4]. The structures were solved with the **ShelXT** (Sheldrick, 2015)[5] solution program using dual methods and by using **Olex2** 1.5 (Dolomanov *et al.*, 2009)[6] as the graphical interface. The models were refined with **ShelXL** 2019/3 (Sheldrick, 2015)[7] using full matrix least squares minimization on $\boldsymbol{F^2}$. All non-hydrogen atoms were refined anisotropically. The positions of the hydrogen atom were calculated geometrically and refined using the riding model. Several structures displayed problems dealing with disorder (disordered ligands or solvent) or twinning. The major employed technique was the split model combined with a series of restraints and constraints. The restraints and constraints are used to get acceptable bond lengths and angles and/or anisotropic behavior. For **5**, the twinning treatment has been used to properly separate the different domains. For **K3NNN**, the mask algorithm (by Olex2) was used to squeeze the solvents completely from the final model.

CCDC deposition numbers 2404451 (**K3NNN**), 2404452 (**1**), 2442062 (**2b**), 2457232 (**2c**), 2392949 (**3**), 2404453 (**4**) and 2404454 (**5**) contain the supplementary crystallographic data for this paper. These data are provided free of charge by the joint Cambridge Crystallographic Data Centre and Fachinformationszentrum Karlsruhe (http://www.ccdc.cam.ac.uk/structures).

S2. Synthesis and Reactivity Studies

Due to the lability of the potassium-bound Et_2O and THF, the stoichiometries and yields of the following reactions were calculated based on the chemical formulas derived from elemental analysis of the isolated compounds.

S2.1. Synthesis of [NNN-3K(Et_2O)$_2$]$_2$ (K3NNN)

Procedure: A 100 mL Schlenk flask equipped with a glass-coated stirring bar was charged with the ligand **H3NNN**[3] (1.500 g, 2.232 mmol, 1.0 equiv.) and 20 mL Et_2O. In a separate vial, KHMDS (1.340 g, 6.717 mmol, 3.0 equiv.) was dissolved in 10 mL of Et_2O and added dropwise to the stirring solution of **H3NNN**. The reaction mixture was stirred for 12 h at room temperature, yielding a dark yellow solution. Upon evaporating to dryness, the solid residue was washed with 10 mL hexane on a porosity 4 glass frit, and recrystallized in 10 mL Et_2O at -40 °C, affording **K3NNN** as bright yellow crystals (880 mg, 47% yield, with the chemical formula of [NNN-3K(Et_2O)$_{0.7}$]$_2$).

Solubility and stability: Once isolated, **K3NNN** is moderately and very soluble in Et_2O and THF, respectively, but only slightly soluble in benzene and toluene (ca. 2 mg/mL). **K3NNN** is very air- and moisiture-sensitive.

^{1}H NMR (400 MHz, THF-d_8, 298 K, **Figure S2**): δ 6.87 (br., 4H, Ar−H), 6.47 (br., 2H, Ar−H), 5.99 (br., 2H, Ar−H), 5.45 (br., 2H, Ar−H), 3.32 (br., 4H, −C**H**(CH_3)$_2$), 1.61 (br., 6H, >C(C**H**$_3$)$_2$), 1.28−0.86 (br., 42H, −C(C**H**$_3$)$_3$ and −CH(C**H**$_3$)$_2$, overlapped with −CH_2C**H**$_3$ of Et_2O). Broadened signals observed in THF-d_8 at 298 K suggests the dynamic behavior of **K3NNN** in solution.

XRD: Single crystals of **K3NNN** suitable for XRD analysis were obtained from a concentrated Et_2O solution at -40 °C, showing the presence of [NNN-3K(Et_2O)$_2$]$_2$ (**Figure S25**). ***Anal. Calcd.*** for NNN-3K(Et_2O)$_{0.7}$, $C_{49.8}H_{69}K_3N_3O_{0.7}$: C, 67.35; H, 7.46; N, 5.01. *Found:* C, 67.33; H, 7.84; N, 4.59. The potassium bound Et_2O was partially lost during the bulk synthesis.

S2.2. Synthesis of [NNN-U(THF)Cl_2K(THF)$_3$]$_2$ (1)

Procedure: A 50 mL Schlenk flask equipped with a glass-coated stirring bar was charged with **K3NNN** (450 mg, 0.2684 mmol, 0.5 equiv.; calcd. as [NNN-3K(Et_2O)$_{0.7}$]$_2$) and 8 mL THF. In a separate vial, UCl_4 (199 mg, 0.5240 mmol, 1.0 equiv.) was dissolved in 6 mL THF and added dropwise to the stirring solution of **K3NNN**. The reaction mixture was stirred for one day at room temperature, yielding a dark green suspension. All volatiles were removed under vacuum. The resultant residue was dissolved in 8 mL Et_2O and filtered over a porosity 4 glass frit. The filtrate was concentrated to ca. 2 mL, to which 4 mL *n*-hexane was added. The resultant solution was placed at -40 °C over two days, affording **1** as green crystals (446.4 mg, 76% yield, with the chemical formula of [NNN-U(THF)Cl_2K]$_2$).

^{1}H NMR (600 MHz, THF-d_8, 298 K, **Figure S3**): δ 9.69 (d, J = 8.0 Hz, 4H, Ar−H of Dipp), 9.18 (t, J = 8.0 Hz, 2H, Ar−H of Dipp), 8.39 (s, 2H, Ar−H of the pincer backbone), 5.25 (s, 2H, Ar−H of the pincer backbone), 2.20−2.00 (m, 4H, −C**H**(CH_3)$_2$), 1.93 (s, 18H, −C(C**H**$_3$)$_3$), 1.66 (s, 12H, −CH(C**H**$_3$)$_2$), 1.19 (s, 6H, >C(C**H**$_3$)$_2$), −0.96 (s, 12H, −CH(C**H**$_3$)$_2$). **^{1}H NMR** (400 MHz, THF-d_8, 233 K, **Figure S4**): δ 8.66 (d, J = 8.1 Hz, 4H, Ar−H of Dipp), 8.54 (t, J = 7.8 Hz, 2H, Ar−H of Dipp), 5.05 (s, 2H, Ar−H of the pincer backbone), 4.30 (s, 2H, Ar−H of the pincer backbone), 2.74−2.09 (br. m, 4H, −C**H**(CH_3)$_2$), 1.26 (s, 18H, −C(C**H**$_3$)$_3$), 1.21 (br. s, 12H, −CH(C**H**$_3$)$_2$), 0.69 (br, s, 6H, >C(C**H**$_3$)$_2$), −0.15 (br. s, 12H, −CH(C**H**$_3$)$_2$).

Stability: No decomposition was observed for complex **1** in the solid state and in THF-d_8 at room temperature over two months and two weeks, respectively. **XRD:** Single crystals of **1** suitable for XRD analysis were obtained from a THF/*n*-hexane solution at -40 °C, showing the presence of the complex [NNN-U(THF)Cl_2K(THF)$_3$]$_2$. ***Anal. Calcd.*** for NNN-U(THF)Cl_2K, $C_{51}H_{70}Cl_2KN_3OU$: C, 56.24; H, 6.48; N, 3.86. *Found:* C, 55.87; H, 6.55; N, 3.71. The potassium bound THF molecules were lost during the bulk synthesis.

S2.3. Synthesis of U(III) pincer complexes 2

S2.3.1. Reduction of 1 with KC_8

A suspension of KC_8 (1.2 mg, 0.0089 mmol, 1.0 equiv.) in THF-d_8 (0.2 mL) was added to a stirring solution of **1** (10.0 mg, 0.0046 mmol, 0.5 equiv.; calcd. as [NNN-U(THF)Cl_2K]$_2$) in THF-d_8 (0.2 mL) at -40 °C, and the formation of graphite was observed. The reaction

mixture was stirred for 2 h at -40 ^{o}C and transferred to a J.Young NMR tube. The ^{1}H NMR spectrum of the reaction mixture showed two sets of resonances, which were assigned to [NNN-U^{III}(THF)]•$(KCl)_n$, **2** and unreacted **1** (**Figure S5** b and **Figure S6** b). The sample was placed at -40 ^{o}C for one day, and ^{1}H NMR was recorded again, showing no evolution of the reaction mixture.

Then, a suspension of KC_8 (1.2 mg, 0.0089 mmol, 1.0 equiv.) in THF-d_8 (0.2 mL) was added to the mixture, resulting in a dark purple suspension. The sample was placed at -40 ^{o}C for 2 h. The ^{1}H NMR spectrum of the reaction mixture showed the complete consumption of **1** (**Figure S5** c and **Figure S6** c). The EPR studies of the reduction mixture filtrate indicate the presence of U(III) species (**Figure S28**).

S2.3.2. Isolation of [NNN-U^{III}(THF)]$_2$•$(KCl)_n$ (2)

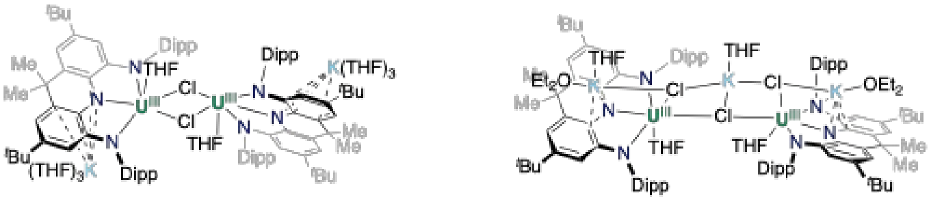

2a **2b**

Figure S1. Structures of the U(III) species **2a** and **2b** identified by XRD analysis.

The binding of variable numbers of bridging KCl molecules complicates the isolation and characterization of the U(III) species **2**. Two different U(III)-ate species, **2a** and **2b**, were isolated from the reduction mixture and characterized by XRD analysis (**Figure S1**).

A suspension of KC_8 (12.4 mg, 0.0917 mmol, 2.0 equiv.) in THF (1.5 mL) was added to a stirring solution of **1** (50.0 mg, 0.0230 mmol, 0.5 equiv.; calcd. as [NNN-U(THF)Cl_2K]$_2$) in THF (2 mL) at -40 ^{o}C. After 3 h stirring at -40 ^{o}C, the suspension was filtered over a pre-chilled porosity 4 glass frit. The dark purple filtrate was concentrated to ca. 1 mL, to which pre-chilled *n*-hexane (4 mL) was added. The resultant solution was placed at -40 ^{o}C over two days, affording a mixture of dark purple and brown microcrystals (31.7 mg). Dark purple single crystals suitable for XRD analysis were obtained from a Et_2O solution of this isolated material at -40 ^{o}C over two days. XRD analysis showed the presence of [NNN-U^{III}(THF)]$_2$$(KCl)_3$$(THF)_2$$(Et_2O)_2$, **2b**. When the solid was recrystallized from a *n*-hexane/THF solution, a mixture of dark purple and brown crystals was obtained over three days at -40 ^{o}C. XRD analysis of a dark brown single crystal showed the presence of [NNN-U^{III}Cl(THF)K$(THF)_3$]$_2$, **2a** (**Figure S26**). However, the poor quality of the single crystal prevents further discussion of the structure of **2a**.

^{1}H NMR of isolated crystals of **2b** (400 MHz, THF-d_8, 233 K, **Figure S7**): δ 26.81 (br.), 22.20, 20.21 (br.), 16.33 (br.), 3.84, 2.69, -10.03 (br.), -12.96 (br.). One set of protons (probably −C**H**$(CH_3)_2$) is missing due to line broadening of species **2**. The species **2** decomposed slowly at -40 ^{o}C but rapidly at room temperature, as monitored by ^{1}H NMR. The ^{1}HNMR spectrum of the reaction mixture containing both 2a and 2b show only on major set of signals.

Elemental analysis for isolated **2b** suggested the presence of co-crystallized KCl. ***Anal. Calcd.*** for [NNN-U(THF)(KCl)]•$(KCl)_2$$(THF)_2$, $C_{59}H_{86}Cl_3K_3N_3O_3U$: C, 56.61; H, 6.44; N, 3.12. *Found:* C, 52.49; H, 6.58; N, 2.87.

S2.3.3. Reaction of 2 with biphenylene

A suspension of KC_8 (2.5 mg, 0.0185 mmol, 2.0 equiv.) in THF-d_8 (0.2 mL) was added to a stirring solution of [NNN-U(THF)Cl_2K] (10.0 mg, 0.0046 mmol, 0.5 equiv.) in THF-d_8 (0.1 mL) at -40 ^{o}C, and the reaction mixture was stirred for 3 h at this temperature [**Figure S8** (a) and **Figure S9** (a)] to yield **2**. Then, a solution of biphenylene (1.4 mg, 0.0092 mmol, 1.0 equiv.) in THF-d_8 (0.2 mL) was added to the reaction mixture, resulting in a color change from dark purple-brown to dark brown. After reacting 3 h at -40 ^{o}C, the ^{1}H NMR spectrum of the reaction mixture [**Figure S8** (b) and **Figure S9** (b)] showed the formation of [NNN-U(THF)(biphenylene)][K$(THF)_5$] (**3**) and the presence of unreacted biphenylene and regenerated **1**, with a 1:1 ratio of **3**:**1** (Note: complex **1** calculated as a monomer; **Figure S10**). The sample was placed at -40 ^{o}C for one day, and ^{1}H NMR was recorded again, showing no evolution of the reaction mixture. These results suggest that the U(III) **2** effects the two-electron reduction of biphenylene, while yielding **1** and **3** in a 1:1 ratio (**Scheme S1**). A suspension of KC_8 (1.2 mg, 0.0089 mmol, 1.0 equiv.) in THF-d_8 (0.1 mL) was added to the mixture of **1** and **3**. After 3 h stirred at -40 ^{o}C, the ^{1}H NMR spectrum of the resultant reaction mixture [**Figure S8** (c) and **Figure S9** (c)] showed a nearly complete conversion of biphenylene and **1** to **3**.

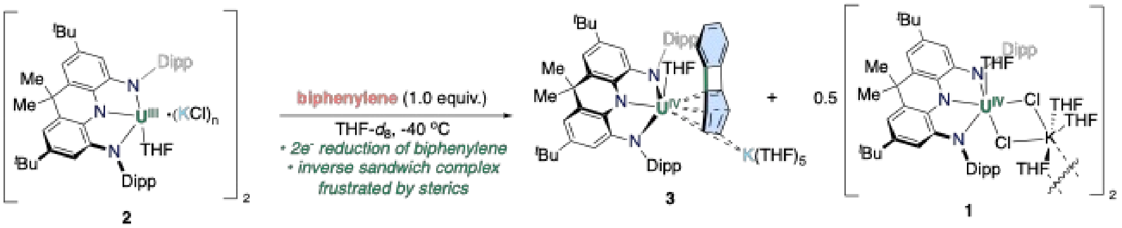

Scheme S1. Reaction of **2** with biphenylene yielding complexes **3** and **1**.

S2.3.4. Reaction of $UI_3(THF)_4$ with K3NNN

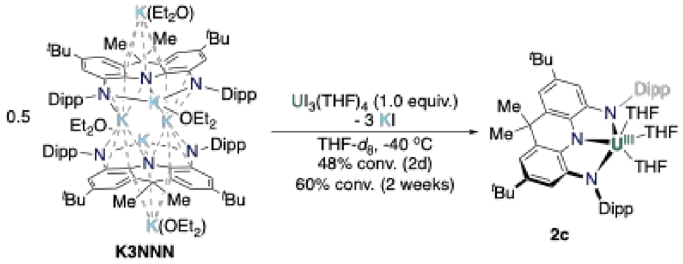

Scheme S2. Reaction of $UI_3(THF)_4$ with **K3NNN** yielding **2c**.

The reaction mixture of $UI_3(THF)_4$ (10.5 mg, 0.0116 mmol, 1.0 equiv.) and **K3NNN** (10.0 mg, 0.0116 mmol; calcd. as [NNN-3K$(Et_2O)_{0.7}]_2$) in 0.6 mL THF-d_8 was stirred at -40 °C, resulting in a dark yellow solution with concomitant formation of white precipitates (presumably KI). The reaction was monitored by ^1H NMR spectroscopy at 233 K after 2 days and 2 weeks, showing the formation of the U(III) **2c** in ca. 48% and 60% conv., respectively, and unreacted **K3NNN** (**Figure S11**). When the reaction mixture was placed at room temperature for 12 h, **2c** decomposed to other unidentifiable species, suggesting **2c** is temperature-sensitive.
Isolation of **2c** from the reaction mixture failed, but dark brown single crystals of **2c** suitable for XRD analysis were obtained from the reaction mixture in THF/*n*-hexane at -40 °C, showing the presence of NNN-U(III)$(THF)_3$ (**Figure S27**).

S2.3.5. Reaction of $U(HMDS)_3$ with H3NNN

The reaction of $U(HMDS)_3$ with **H3NNN** was attempted but without success probably due to the bulk of the **H3NNN** ligand. The reaction proceeds slowly, requires high temperature and does not lead to the desired U(III) complex.

S2.4. Synthesis of [NNN-U(THF)(biphenylene)][K$(THF)_5$] (3)

Procedure 1: A 6 mL vial with a glass-coated stirring bar was charged with **1** (50 mg, 0.0230 mmol, 0.5 equiv.; calcd. as [NNN-U(THF)$Cl_2K]_2$), biphenylene (7 mg, 0.0460 mmol, 1.0 equiv.) and 2 mL THF and chilled to -40 °C. To this solution a pre-chilled KC_8 suspension (12.4 mg, 0.0917 mmol, 2.0 equiv., in 1 mL THF) was added, resulting in a dark brown suspension, with concomitant formation of graphite. After 12 h stirring at -40 °C, the suspension was filtered over a pre-chilled porosity 4 glass frit. The filtrate was concentrated to ca. 1.5 mL and pre-chilled *n*-hexane (1.5 mL) was added. The resultant solution was placed at -40 °C overnight, affording dark brown crystals of **3** (44.9 mg, 84 % yield, with the chemical formula of [NNN-U(THF)(biphenylene)][K]).

Procedure 2: A 15 mL vial with a glass-coated stirring bar was charged with **1** (150 mg, 0.0689 mmol, 0.5 equiv.; calcd. as [NNN-U(THF)$Cl_2K]_2$) and 3 mL THF and chilled to -40 °C. To this solution a pre-chilled KC_8 suspension (37.2 mg, 0.2751 mmol, 2.0 equiv., in 1.5 mL THF) was added, resulting in a dark purple suspension, with concomitant formation of graphite. The reaction mixture was stirred for 3 h at -40 °C. Biphenylene (21 mg, 0.1380 mmol, 1.0 equiv.) in 1 mL pre-chilled THF was added to the resultant mixture, yielding a dark brown suspension. The reaction mixture was stirred for 3 h at -40 °C, to which another portion of pre-chilled KC_8 suspension (18.6 mg, 0.1376 mmol, 1.0 equiv., in 1 mL THF) was added. After 12 h stirring at -40 °C, the suspension was filtered over a pre-chilled porosity 4 glass frit. The filtrate was concentrated to ca. 2 mL and pre-chilled *n*-hexane (4 mL) was added. The resultant solution was placed at -40 °C over two days, affording dark brown needles of **3** (122.6 mg, 76% yield, with the chemical formula of [NNN-U(THF)(biphenylene)][K]).

^{1}H NMR (400 MHz, THF-d_8, 233 K, **Figure S12** and **Figure S13**): δ 20.95 (s, 2H), 7.59 (s, 2H), 5.86 (s, 2H), 3.40 (t, *J* = 7.4 Hz, 2H, Ar−H of Dipp), 2.93 (s, 12H, −CH(C**H**$_3$)$_2$), 2.17 (d, *J* = 8.6 Hz, 4H, Ar−H of Dipp), 0.87 (s, 18H, −C(C**H**$_3$)$_3$, overlapped with −C**H**$_3$ of *n*-hexane), 0.19 (s, 2H), −3.08 (br. s, 12H, −CH(C**H**$_3$)$_2$), −29.28 (s, 2H), −137.15 (s, 2H). The ^{1}H NMR signals at δ 20.95, 7.59, 5.86, 0.19, −29.28 and −137.15 can be assigned to the protons of biphenylene and the acridine moiety of the pincer backbone, suggesting the inequivalence of two phenyl rings of the bound biphenylene, consistent with the solid-state structure of **3**. −C**H**(CH$_3$)$_2$ and >C(C**H**$_3$)$_2$ are not found probably due to line broadening. **^{1}H NMR** (400 MHz, THF-d_8, 298 K, **Figure S14**): δ 15.80 (br.), 6.25 (s, 2H), 4.61 (t, *J* = 7.4 Hz, 2H, Ar−H of Dipp), 3.85 (d, *J* = 7.4 Hz, 4H, Ar−H of Dipp), 2.16 (s, 12H, −CH(C**H**$_3$)$_2$), 1.51 (s, 2H), 0.91 (s, 18H, −C(C**H**$_3$)$_3$), −0.56 (br.), −18.45 (br.), −99.56 (br. s, 2H).

Stability: No decomposition products were observed for complex **3** in THF-d_8 at -40 °C over one week by ^{1}H NMR spectroscopy. Complex **3** decomposed very slowly in solution at room temperature, yielding [NNN-U(THF)(2,2'-biphenyl)][K(THF)$_2$] (**4**) as the major product (**Figure S15**). This process was monitored over one month, with approx. 84% conversion of **3** reached. **XRD:** Single crystals of **3** suitable for XRD analysis were obtained from a THF/*n*-hexane solution at -40 °C over two days, showing the presence of [NNN-U(THF)(biphenylene)][K(THF)$_5$]. ***Anal. Calcd.*** for [NNN-U(THF)(biphenylene)][K], $C_{63}H_{78}KN_3OU$: C, 64.65; H, 6.72; N, 3.59. *Found:* C, 64.22; H, 6.98; N, 3.26. The potassium bound THF molecules were lost during the bulk synthesis.

S2.5. Synthesis of [NNN-U(THF)(2,2'-biphenyl)][K(THF)$_2$] (4)

Procedure: A 50 mL Schlenk flask with a glass-coated stirring bar was charged with **3** (150.0 mg, 0.1282 mmol; calcd. as [NNN-U(THF)(biphenylene)][K]) and 4 mL THF. The solution was stirred at 80 °C for 3 h, resulting in a gradual color change from dark brown to orange-red. The resultant reaction mixture was concentrated to ca. 2 mL and 3 mL *n*-hexane was added. The resultant solution was placed at -40 °C, affording [NNN-U(THF)(2,2'-biphenyl)][K(THF)$_2$] (**4**) as a brick-red microcrystalline solid (121.1 mg, 72 % yield).
^{1}H NMR analysis of the reaction mixture (using 8 mg [NNN-U(THF)(biphenylene)][K], independently set up in THF-d_8) showed the full consumption of **3** in 2 h, with concomitant formation of **4** (**Figure S16**).

^{1}H NMR (400 MHz, THF-d_8, 233 K, **Figure S17** and **Figure S18**): δ 203.00 (s, 1H, Ar−H of biphenyl), 77.85 (s, 1H, Ar−H of biphenyl), 58.82 (s, 1H, Ar−H of biphenyl), 51.04 (s, 1H, Ar−H of biphenyl), 39.17 (s, 2H), 32.38 (s, 3H, >C(C**H**$_3$)$_2$), 31.20 (s, 2H), 16.48 (s, 1H, Ar−H of biphenyl), 12.43 (s, 3H, >C(C**H**$_3$)$_2$), 11.45 (s, 6H, −CH(C**H**$_3$)$_2$), 2.36 (s, 18H, −C(C**H**$_3$)$_3$), 1.43 (s, 6H, −CH(C**H**$_3$)$_2$), 1.03 (s, 1H, Ar−H of biphenyl), −9.64 (s, 2H), −10.70 (s, 2H), −13.30 (s, 2H), −17.12 (s, 2H), −20.53 (s, 1H, Ar−H of biphenyl), −21.43 (s, 6H, −CH(C**H**$_3$)$_2$), −35.04 (s, 1H, Ar−H of biphenyl), −44.58 (s, 6H, −CH(C**H**$_3$)$_2$), −113.41 (s, 2H). **^{1}H NMR** (400 MHz, THF-d_8, 298 K, **Figure S19**): δ 23.23 (s, 2H), 1.63 (s, 18H, −C(C**H**$_3$)$_3$), −5.02 (t, *J* = 8.0 Hz, 2H, Ar−H of Dipp), −8.22 (s, 2H). Only 4 sets of protons are found for complex **4** at 298 K due to dynamic behaviour.

Stability: No decomposition was observed for complex **4** in the THF-d_8 solution at room temperature over one week. Complex **4** decomposed slowly at 80 °C and persisted after two days at this temperature. One of the decomposition byproducts was identified as biphenyl. **XRD:** Single crystals of **4** suitable for XRD analysis were obtained from a THF/*n*-hexane solution at -40 °C over two days, showing the presence of [NNN-U(THF)(2,2'-biphenyl)][K(THF)$_2$]. ***Anal. Calcd.*** for [NNN-U(THF)(2,2'-biphenyl)][K(THF)$_2$], $C_{71}H_{94}KN_3O_3U$: C, 64.87; H, 7.21; N, 3.20. *Found:* C, 64.71; H, 7.33; N, 3.00.

S2.6. Synthesis of [NNN-U(THF)$_2$(fluorenone)][K(THF)$_4$] (5)

Procedure: A J. Young NMR tube was charged with complex **4** (49 mg, 0.0373 mmol) and 0.5 mL THF and connected to a Schlenk line. The solution was degassed by three cycles of freeze-pump-thawing and 1 atm CO was introduced to the reaction mixture at -78 °C, resulting in an immediate color change from orange-red to dark red. The reaction mixture was concentrated to ca. 0.8 mL and 1.6 mL *n*-hexane was added. The resultant solution was placed at -40 °C, yielding dark red crystals of **5** (35.8 mg, 76%, with the chemical formula of [NNN-U(THF)$_2$(fluorenone)][K]).
^{1}H NMR analysis of the reaction mixture immediately after adding CO (using 8 mg complex **4**, independently set up in THF-d_8, **Figure S20**) showed the full conversion from **4** to **5**.

^{1}**H NMR** (400 MHz, THF-d_8, 233 K, **Figure S21** and **Figure S22**): δ 45.51 (br. s), 37.63 (s, 2H), 22.59 (br. s), 11.60 (br. s), 8.24 (d, *J* = 9.8 Hz, 2H, Ar−H of fluorenone), 2.26 (s, 18H, −C(C**H**$_3$)$_3$), −1.98 (s, 2H), −2.41 (t, *J* = 9.8 Hz, 2H, Ar−H of fluorenone), −4.71 (br. s), −7.99 (s, 2H), −12.32 (br. s), −15.44 (s, 2H), −17.91 (br. s), −31.07 (br. s), −37.37 (br. s), −80.12 (br. s), −142.13 (br. s). ^{1}**H NMR** (400 MHz, THF-d_8, 298 K, **Figure S23** and **Figure S24**): δ 29.33 (s, 2H), 26.35 (br. s), 8.84 (d, *J* = 8.6 Hz, 2H, Ar−H of fluorenone), 8.30 (br. s, 2H), 1.83 (s, 18H, −C(C**H**$_3$)$_3$), 0.40 (d, *J* = 8.6 Hz, 2H, Ar−H of fluorenone), 0.19 (t, *J* = 8.0 Hz, 2H, Ar−H of fluorenone), −3.92 (t, *J* = 8.0 Hz, 2H, Ar−H of fluorenone), −9.03 (br. s), −10.88 (s, 2H), −13.40 (br. s). Some protons cannot be found for complex **5** at 298 K due to dynamic behaviour.

Stability: Complex **5** is stable in THF-d_8 at room temperature for at least one week. **XRD:** Single crystals of **5** suitable for XRD analysis were obtained from a THF/*n*-hexane solution at -40 ^{o}C over two days, showing the presence of [NNN-U(THF)$_2$(fluorenone)][K(THF)$_4$]. ***Anal. Calcd.*** for [NNN-U(THF)$_2$(fluorenone)][K], $C_{68}H_{86}KN_3O_3U$: C, 64.28; H, 6.82; N, 3.31. *Found:* C, 64.17; H, 6.94; N, 3.22. The potassium bound THF molecules were lost during the bulk synthesis.

S3. NMR Spectroscopic Data

S3.1. NMR spectrum of isolated K3NNN

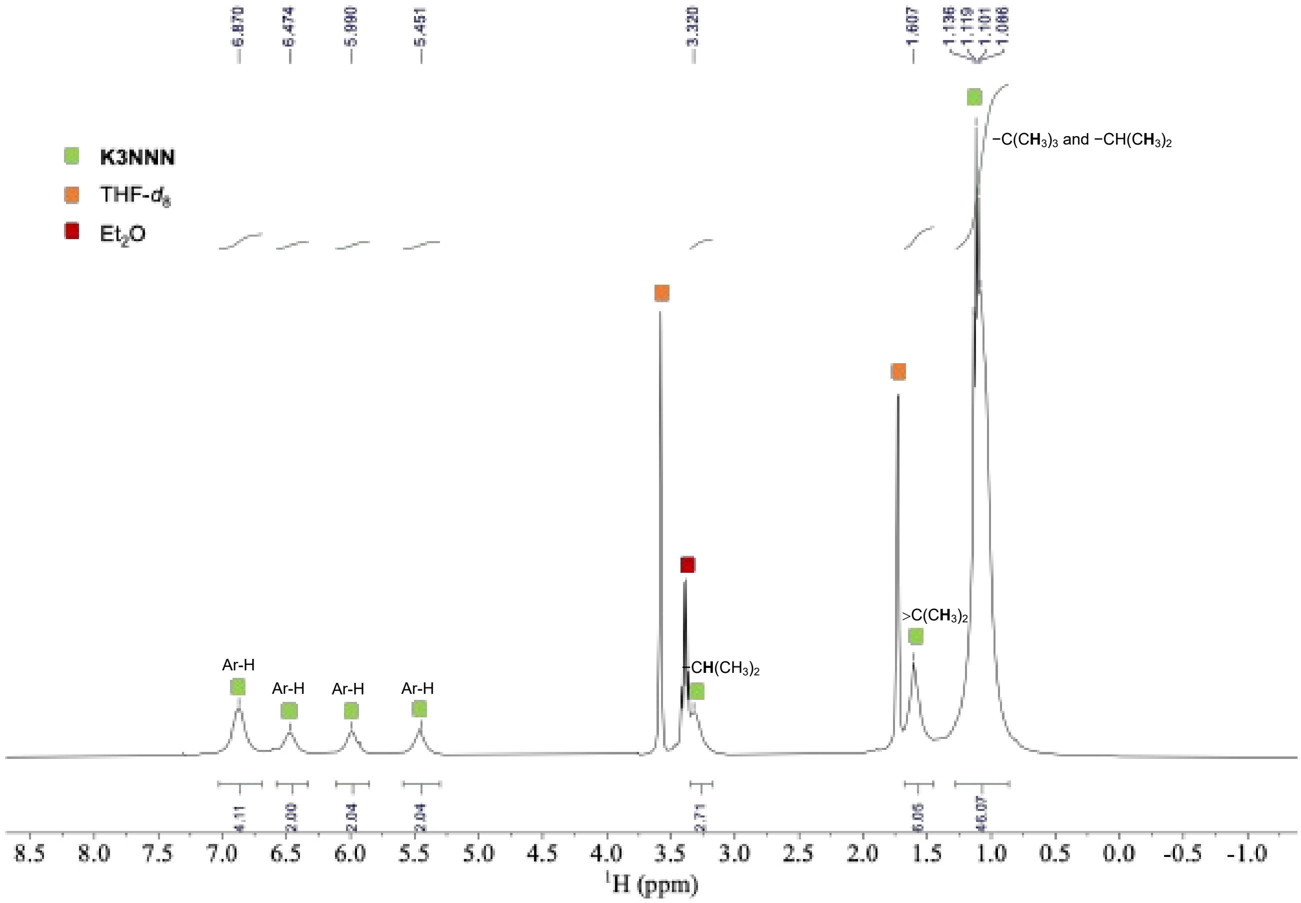

Figure S2. ^1H NMR spectrum (400 MHz, THF-d_8, 298 K) of isolated **K3NNN**.

S3.2. NMR spectra of isolated [NNN-U(THF)Cl$_2$K(THF)$_3$]$_2$ (1)

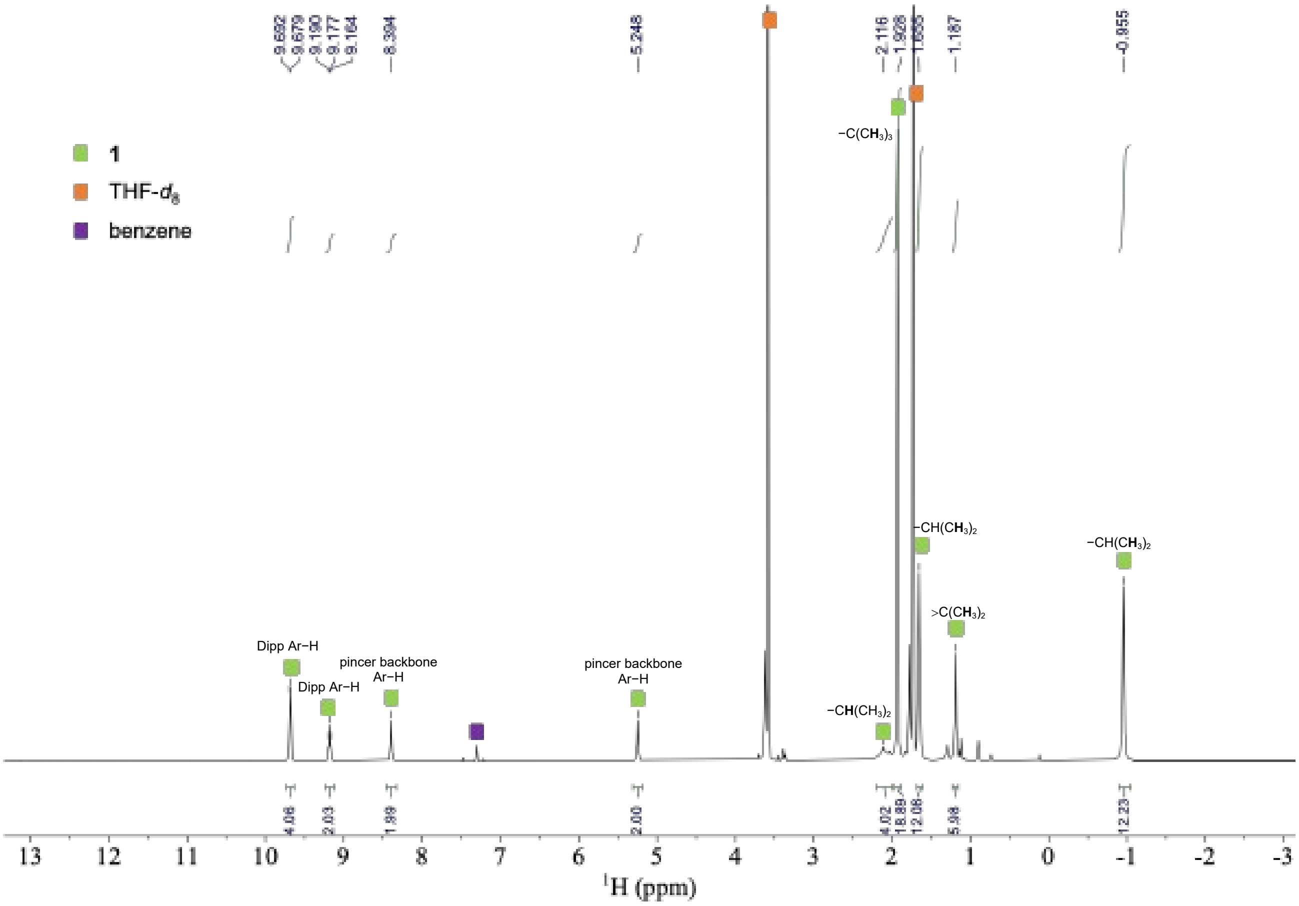

Figure S3. ^{1}H NMR spectrum (600 MHz, THF-d_8, 298 K) of isolated **1**.

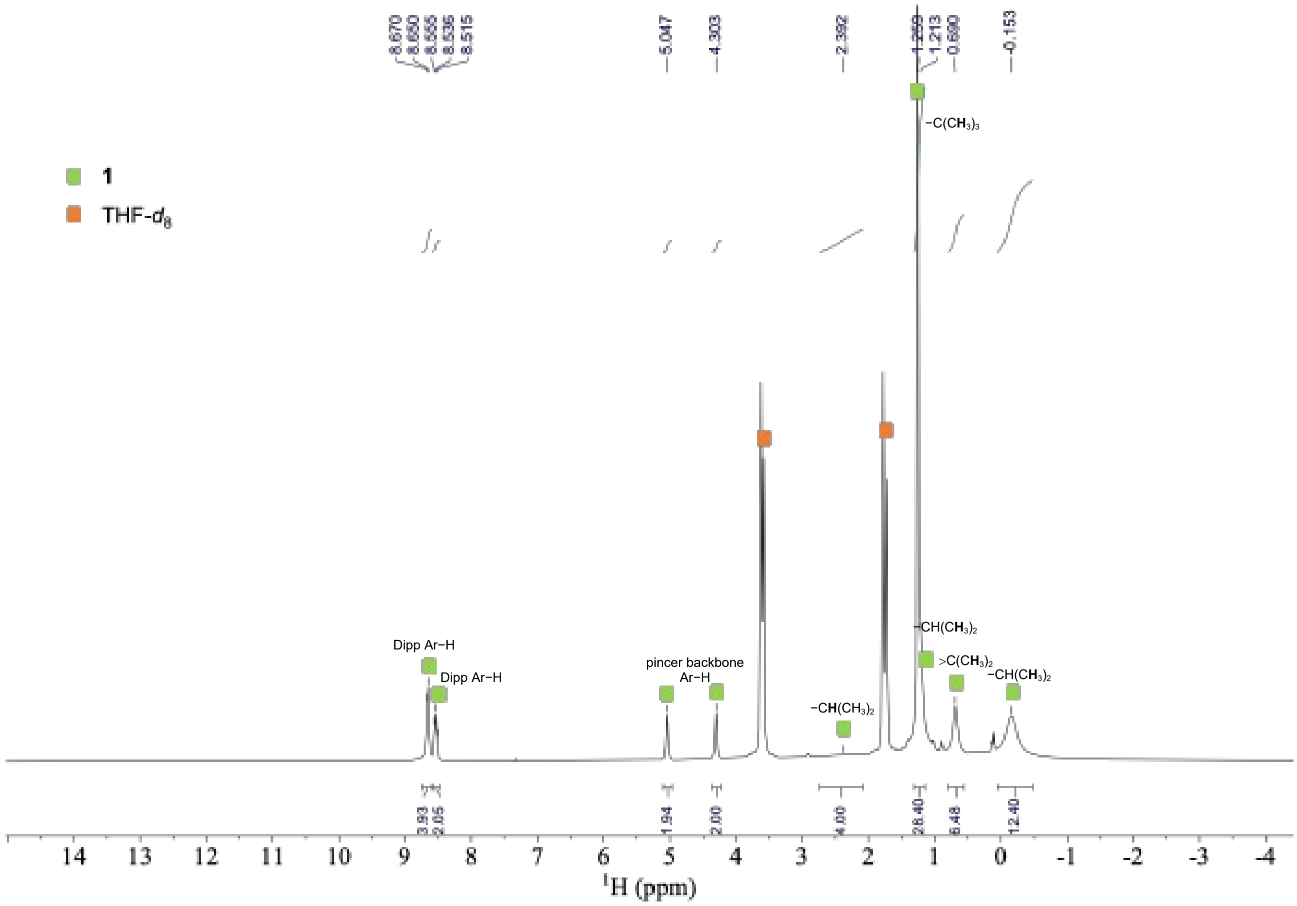

Figure S4. ^{1}H NMR spectrum (400 MHz, THF-d_8, 233 K) of isolated **1**.

S3.3. NMR spectra of the U(III) 2

S3.3.1. Formation of the U(III) 2 by the reduction of 1 with KC_8

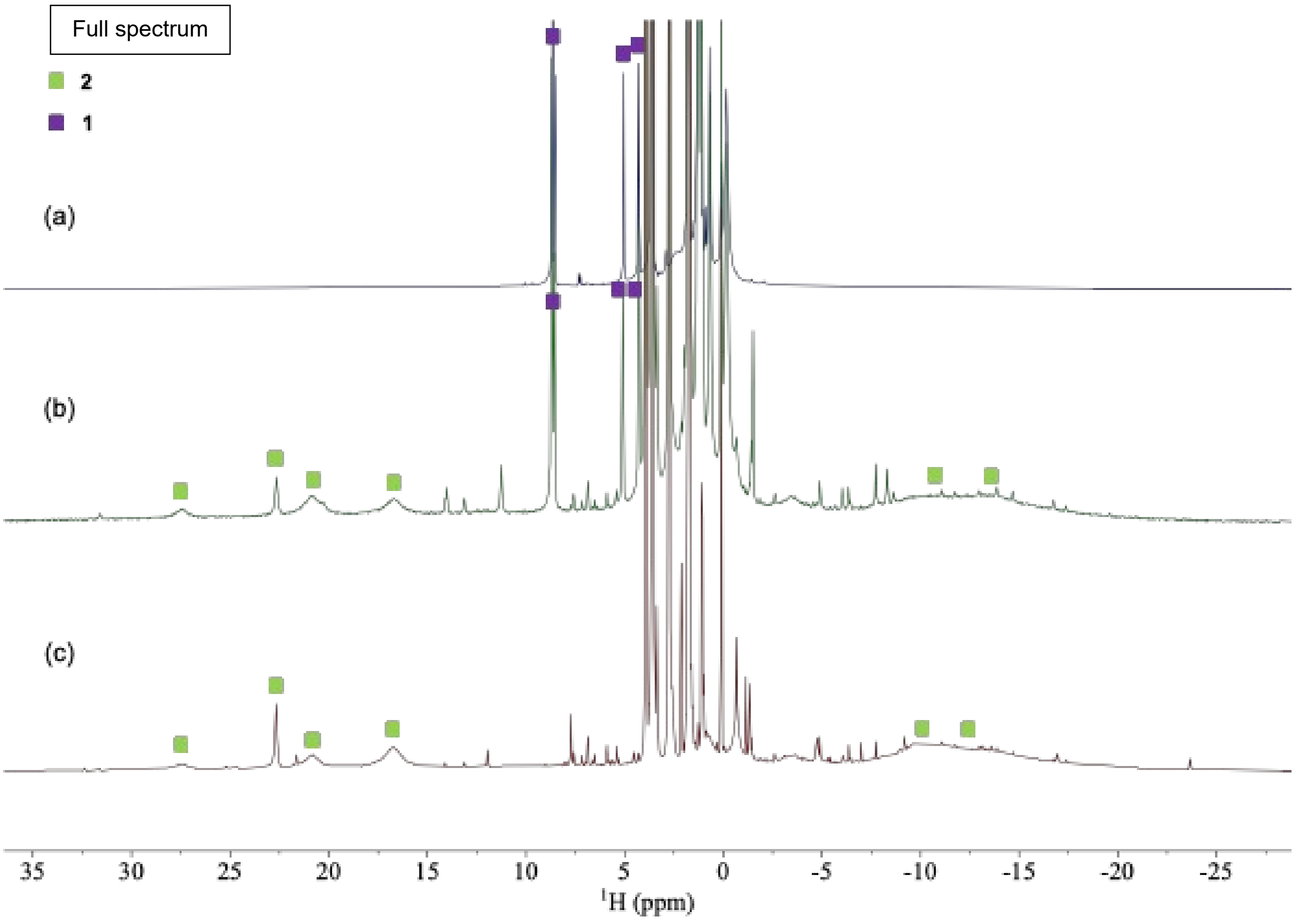

Figure S5. ^{1}H NMR spectra (400 MHz, THF-d_8, 233 K) of the reaction mixture obtained after addition of 1.0−2.0 equiv. of KC_8 to **1** at -40°C (a) before (b) **1** and 1.0 equiv. of KC_8 after 2 h (c) **1** and 2.0 equiv. of KC_8 after 2 h.

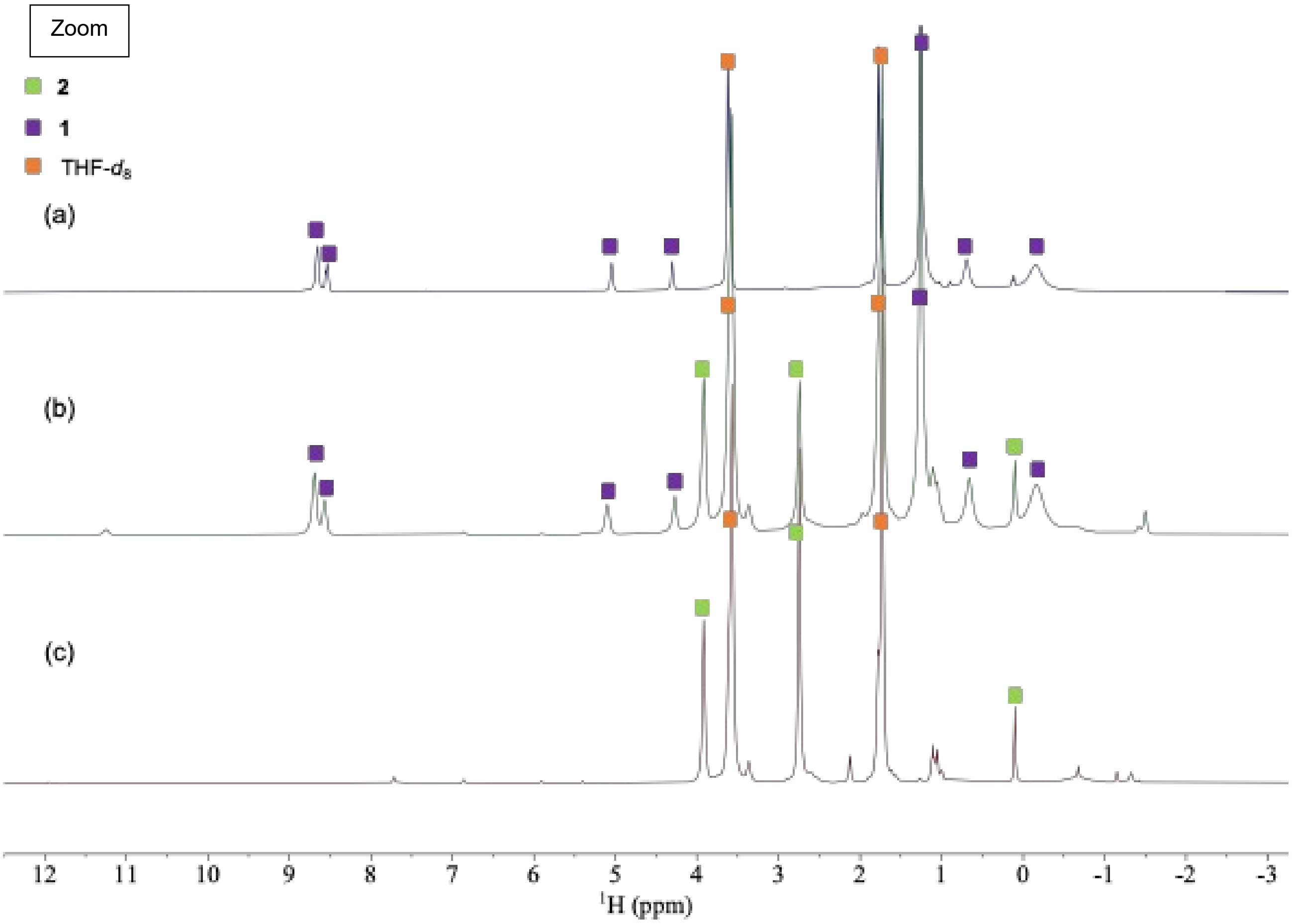

Figure S6. Zoom of the diamagnetic region in **Figure S5**, showing the gradual disappearance of **1**.

S3.3.2. NMR spectrum of isolated U(III) 2b

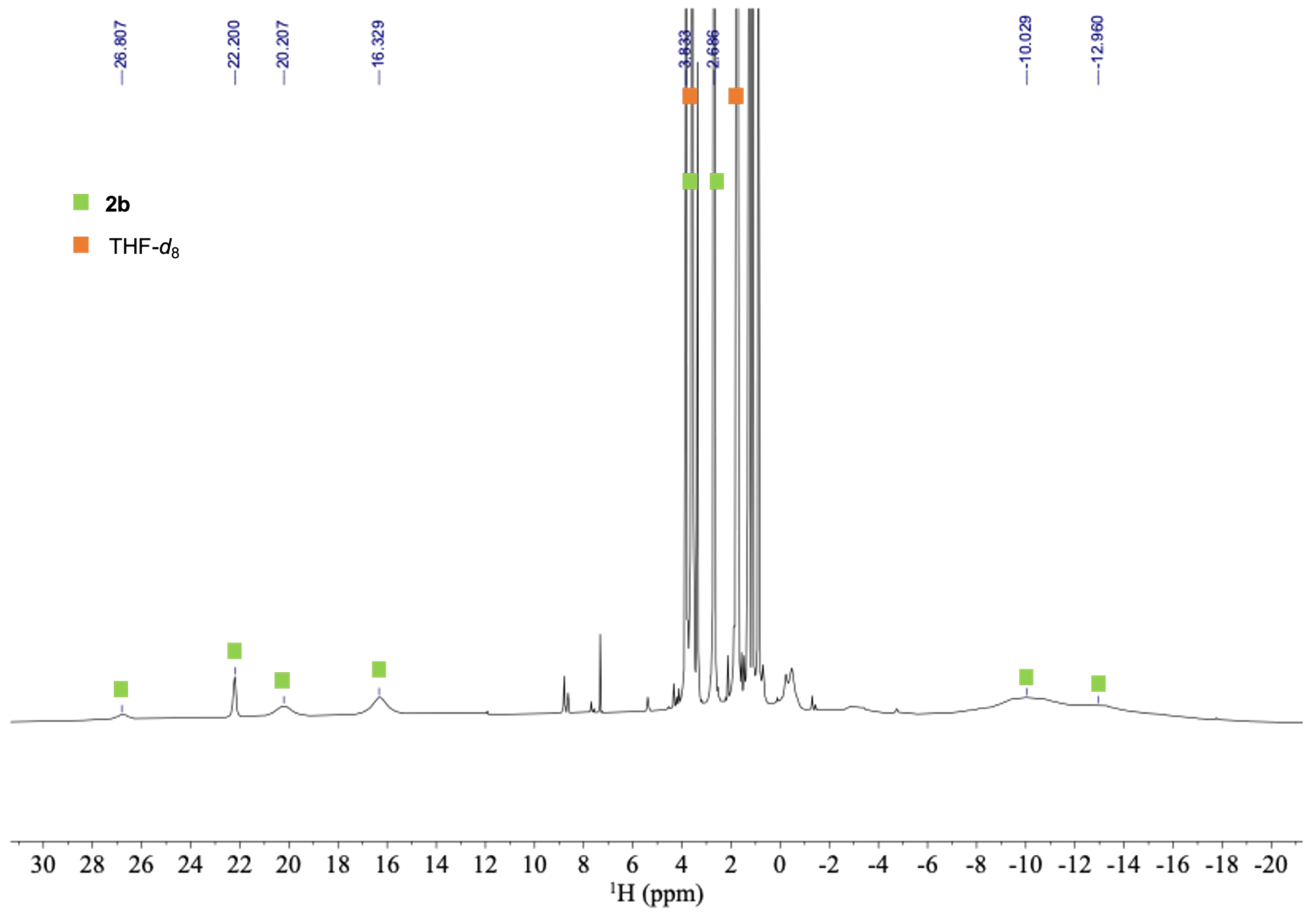

Figure S7. ^{1}H NMR spectrum (400 MHz, THF-d_8, 233 K) of the single crystals of **2b**.

S3.3.3. Reaction of 2 with biphenylene

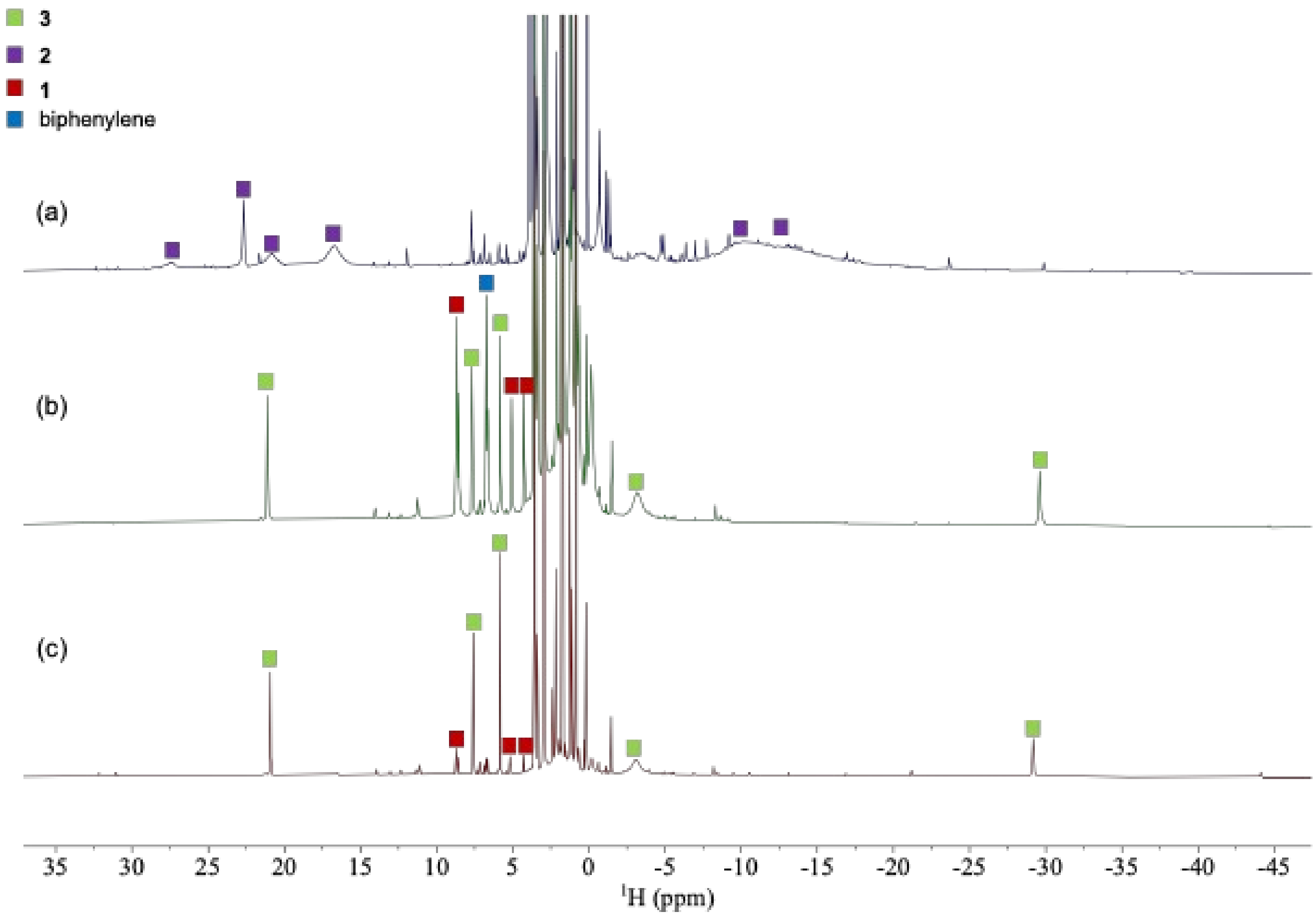

Figure S8. ^1H NMR spectra (400 MHz, THF-d_8, 233 K) of the reaction mixture obtained at -40°C: (a) 3 h after addition of 2.0 equiv. of KC_8 to **1** to yield **2**; (b) 3 h after addition of 1.0 equiv. of biphenylene to the mixture of (a); (c) 3h after addition of 1.0 equiv. of KC_8 to the mixture of (b).

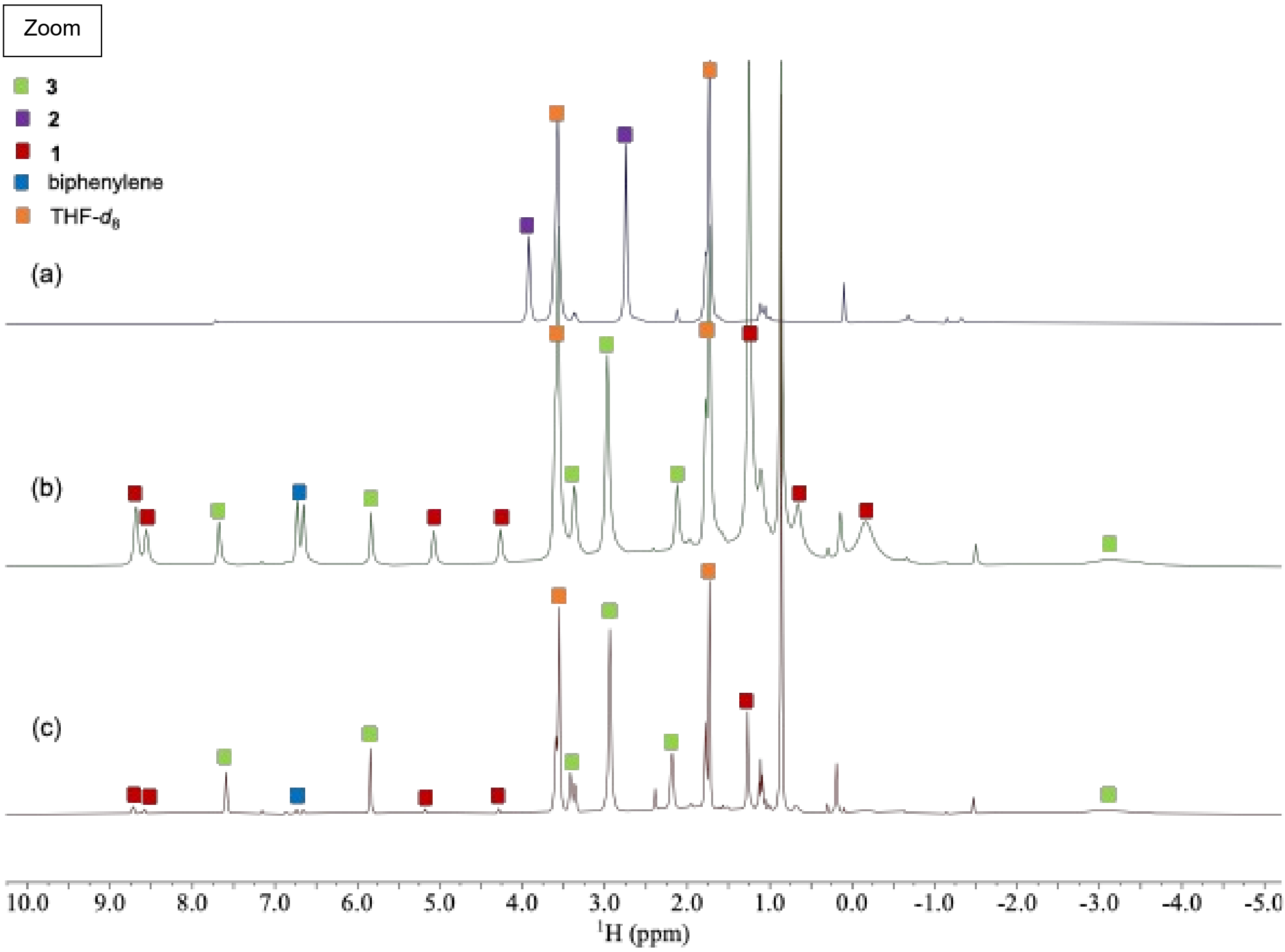

Figure S9. Zoom of the diamagnetic region in **Figure S8**.

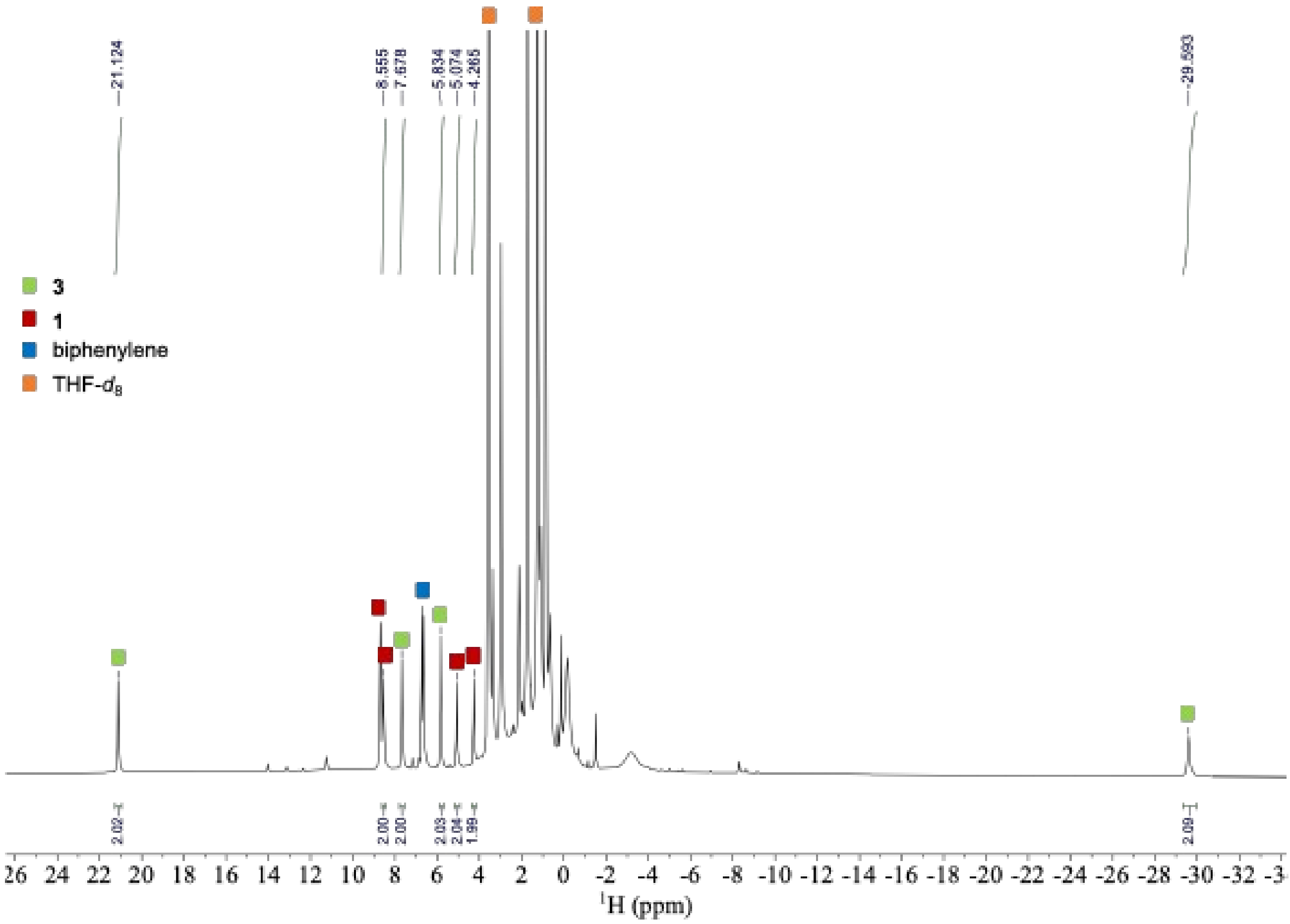

Figure S10. ^{1}H NMR spectra (400 MHz, THF-d_8, 233 K): 3 h after addition of 1.0 equiv. of biphenylene to the *in-situ* generated U(III) **2** at -40 ^{o}C [*i.e.* **Figure S8** (b)], showing the formation of **1** and **3** in a ratio of 1:1 (complex **1** calculated as monomer).

S3.3.4. Reaction of $UI_3(THF)_4$ with K3NNN

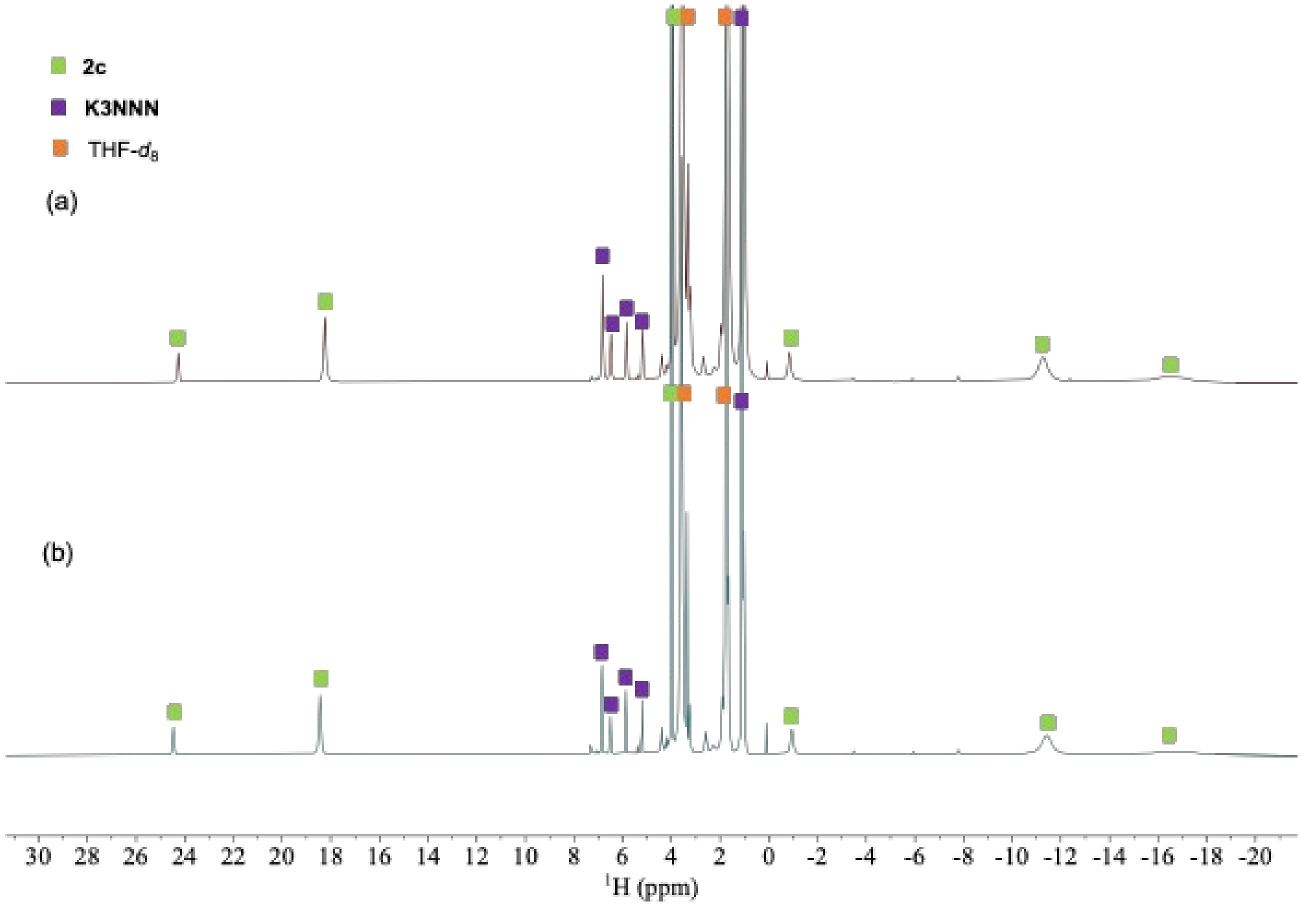

Figure S11. ^{1}H NMR spectra (400 MHz, THF-d_8, 233 K) of the reaction mixture of $UI_3(THF)_4$ and **K3NNN** after (a) 2 d; (b) 2 weeks at -40 ^{o}C, showing the slow formation of the U(III) **2c**.

S3.4. NMR spectra of [NNN-U(THF)(biphenylene)][K(THF)$_5$] (3)

S3.4.1. NMR spectra of isolated [NNN-U(THF)(biphenylene)][K(THF)$_5$] (3)

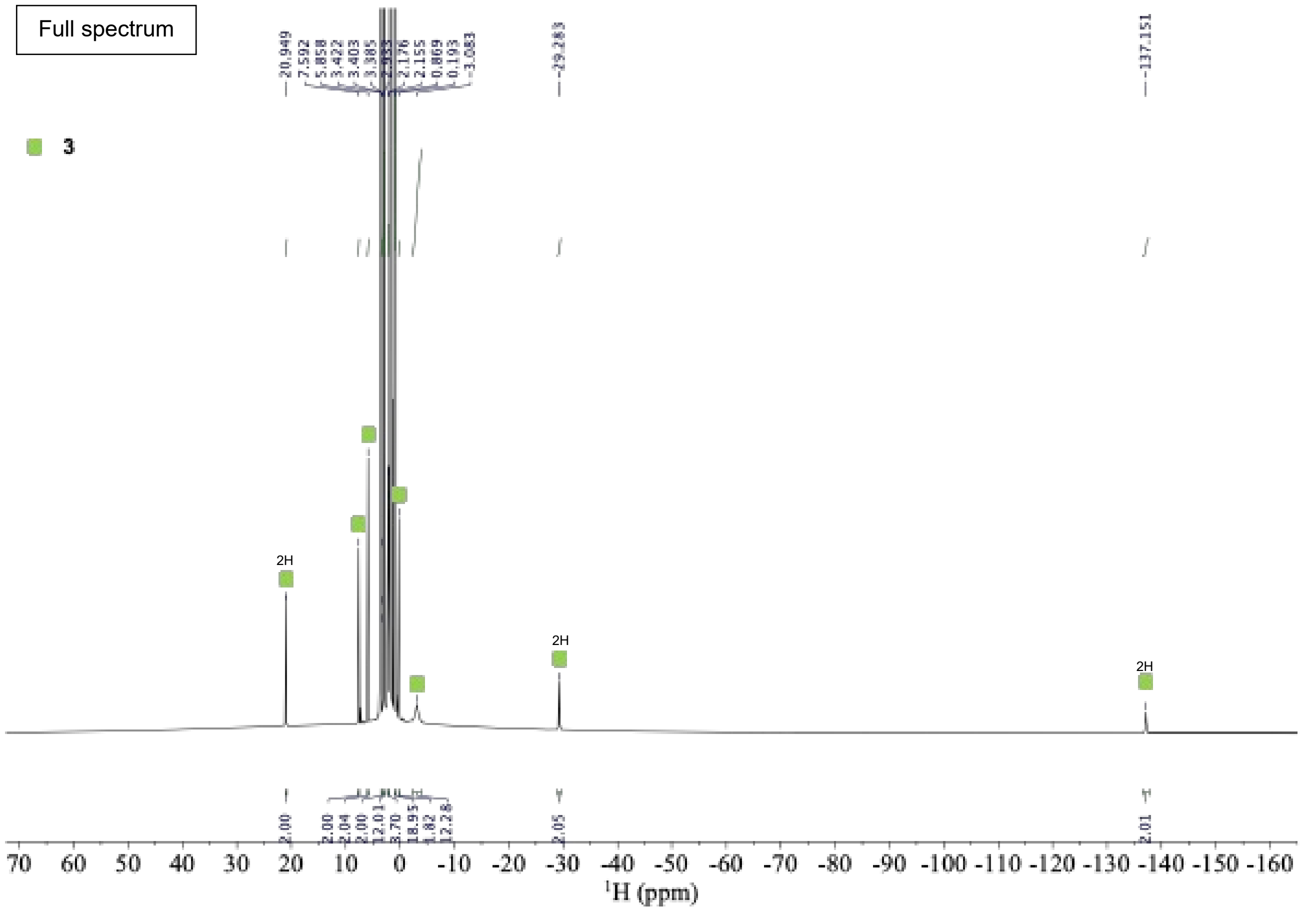

Figure S12. ^{1}H NMR spectrum (400 MHz, THF-d_8, 233 K) of isolated **3**.

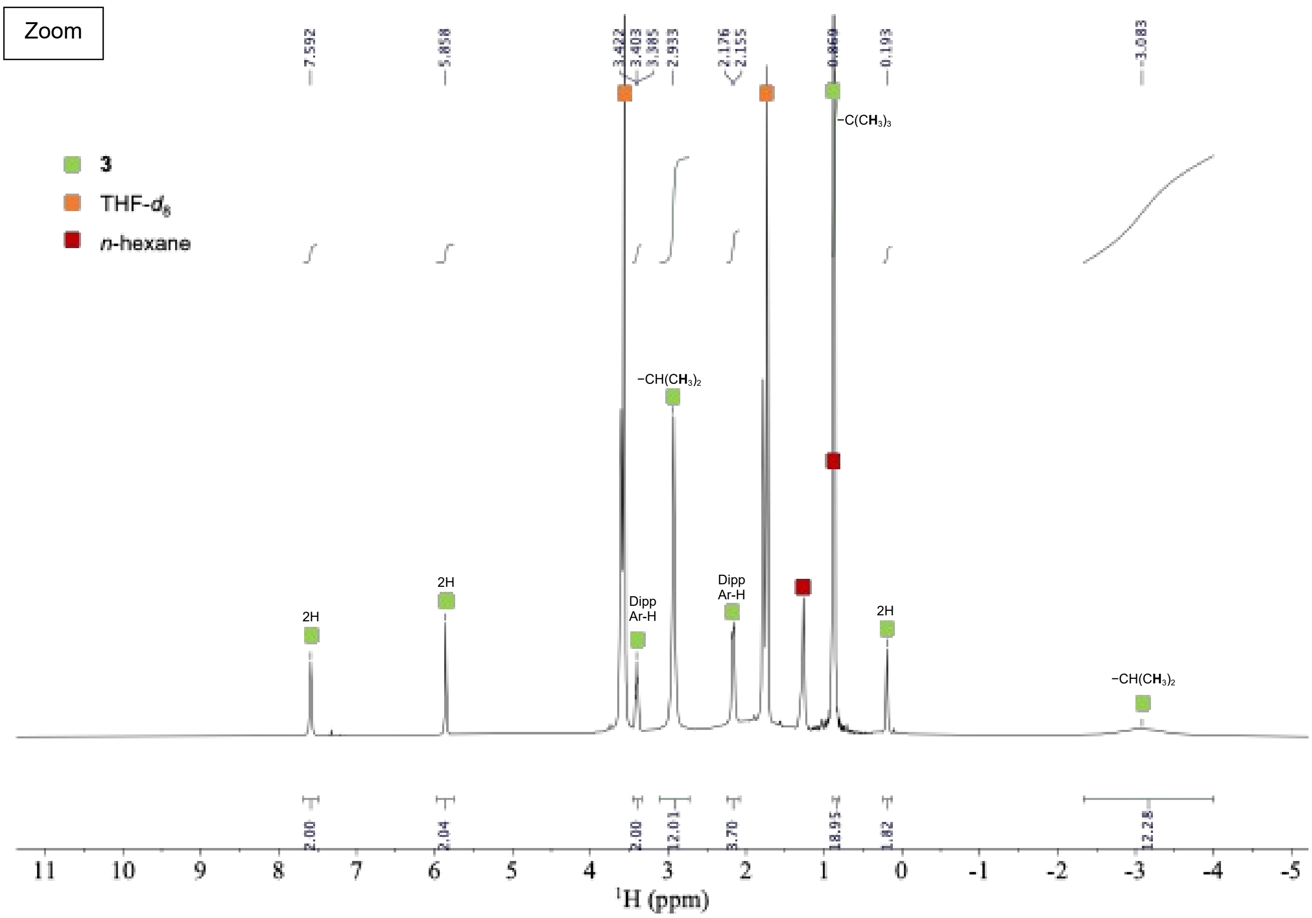

Figure S13. Zoom of the diamagnetic region in **Figure S12**.

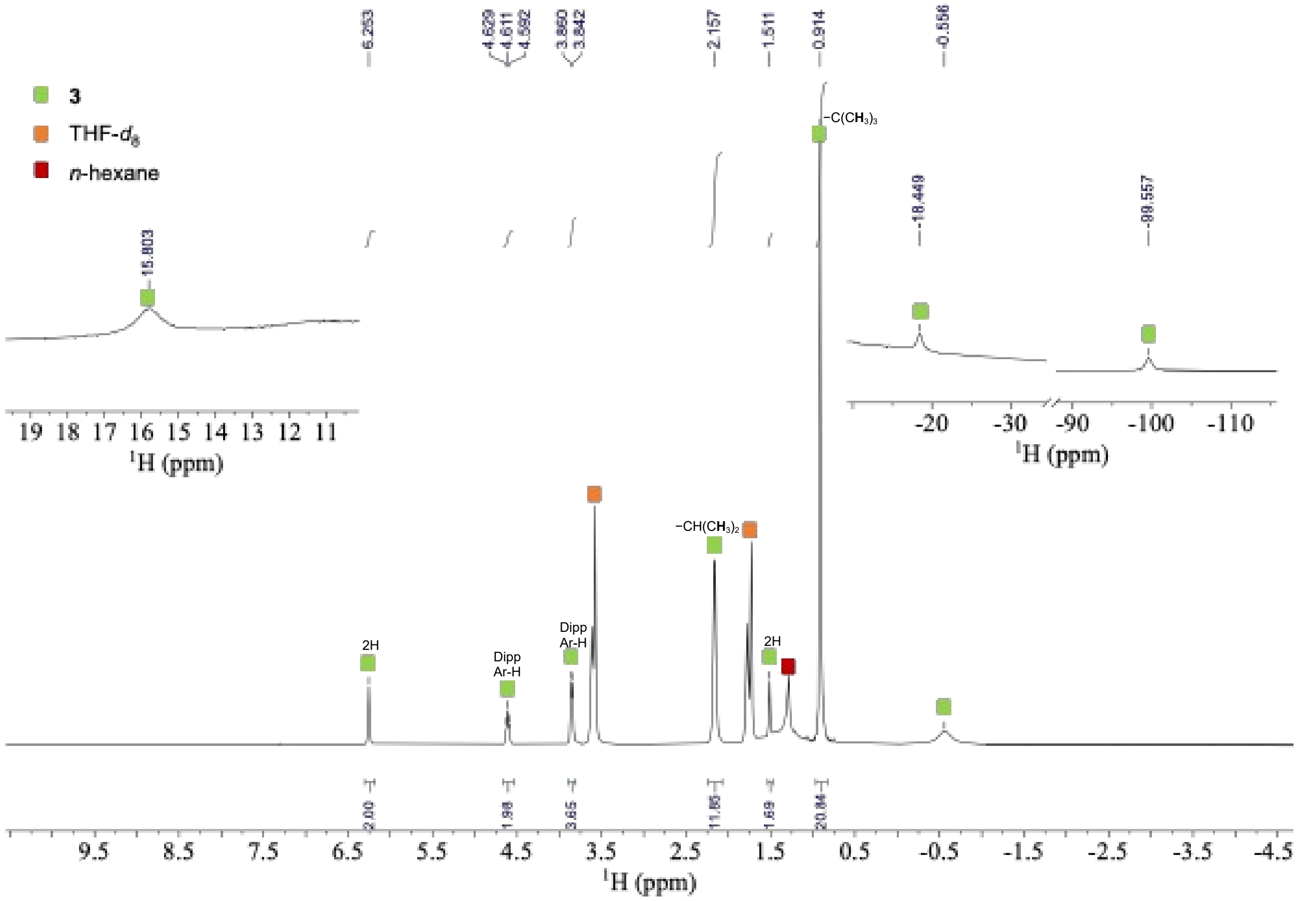

Figure S14. ^{1}H NMR spectrum (400 MHz, THF-d_8, 298 K) of isolated **3**.

S3.4.2. NMR spectra of 3 at room temperature over one month

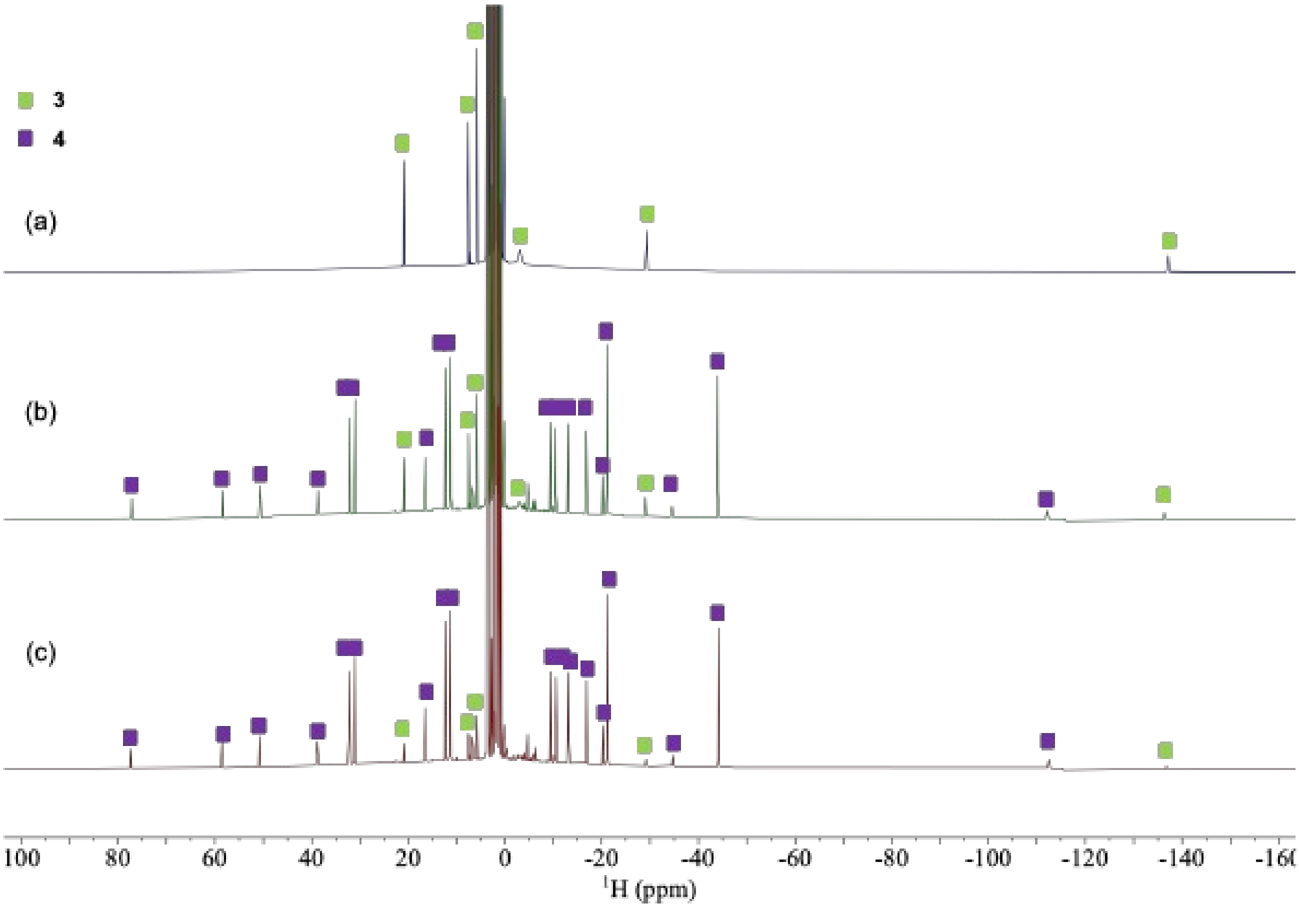

Figure S15. ^{1}H NMR spectrum (400 MHz, THF-d_8, 233 K) of isolated **3** at room temperature, (a) immediately; (b) after two weeks; (c) after one month.

S3.4.3. NMR spectra of 3 at 80 °C over 2 h

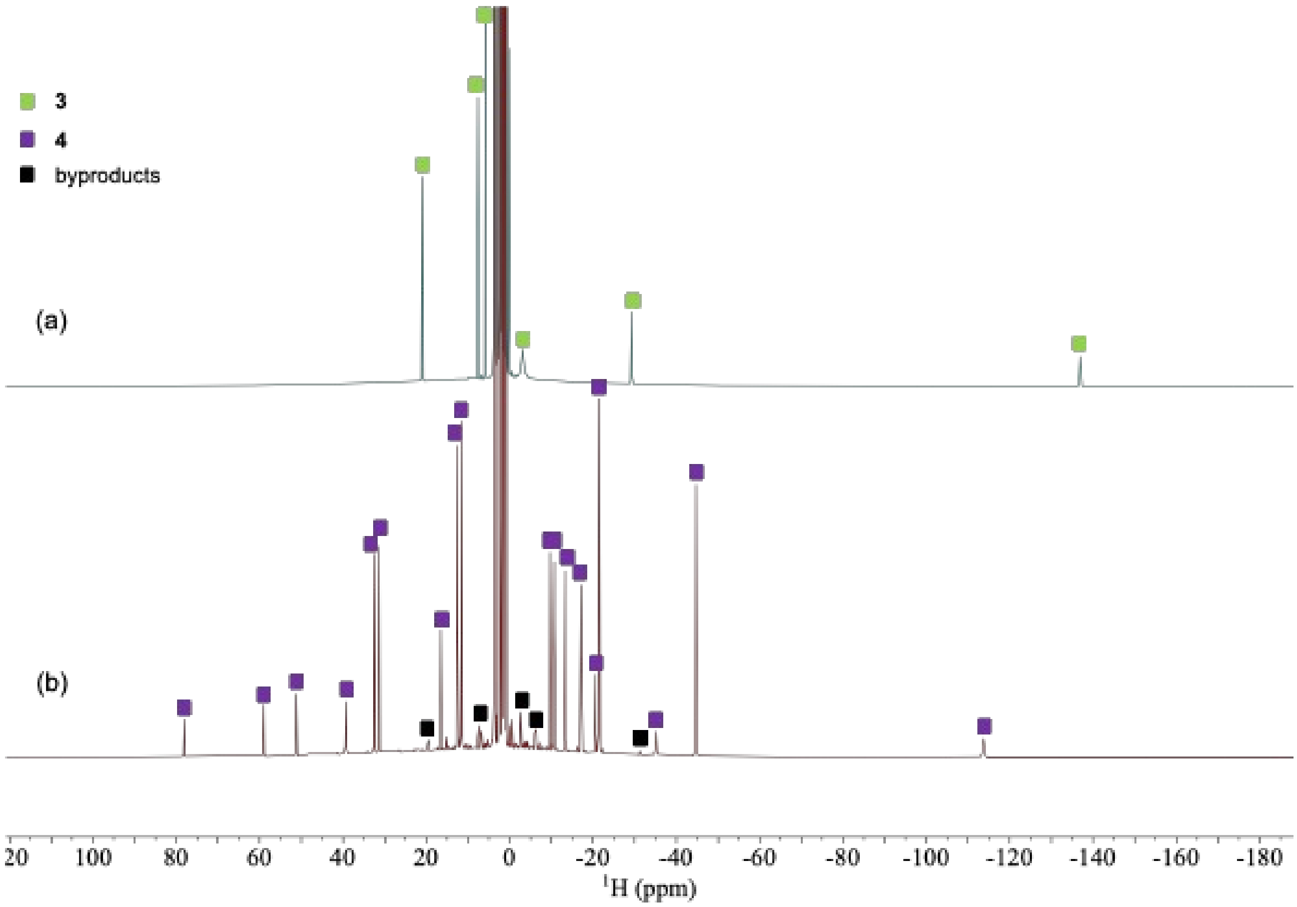

Figure S16. ^{1}H NMR spectrum (400 MHz, THF-d_8, 233 K) of **3**, (a) before; (b) after 2 h at 80 °C.

S3.5. NMR spectra of [NNN-U(THF)(2,2'-biphenyl)][K(THF)$_2$] (4) and its reaction with CO

S3.5.1. NMR spectra of isolated 4

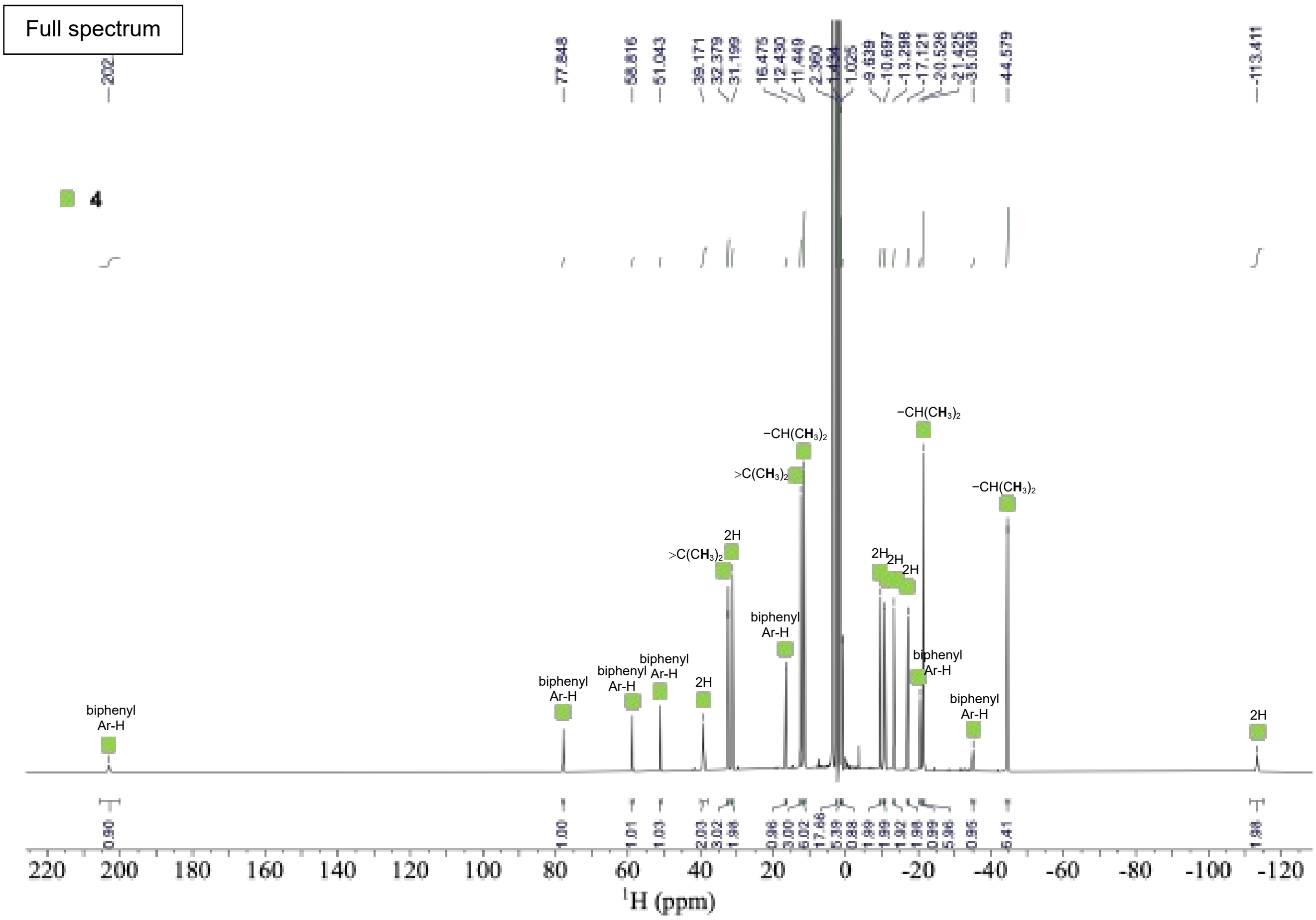

Figure S17. ^{1}H NMR spectrum (400 MHz, THF-d_8, 233 K) of isolated **4**.

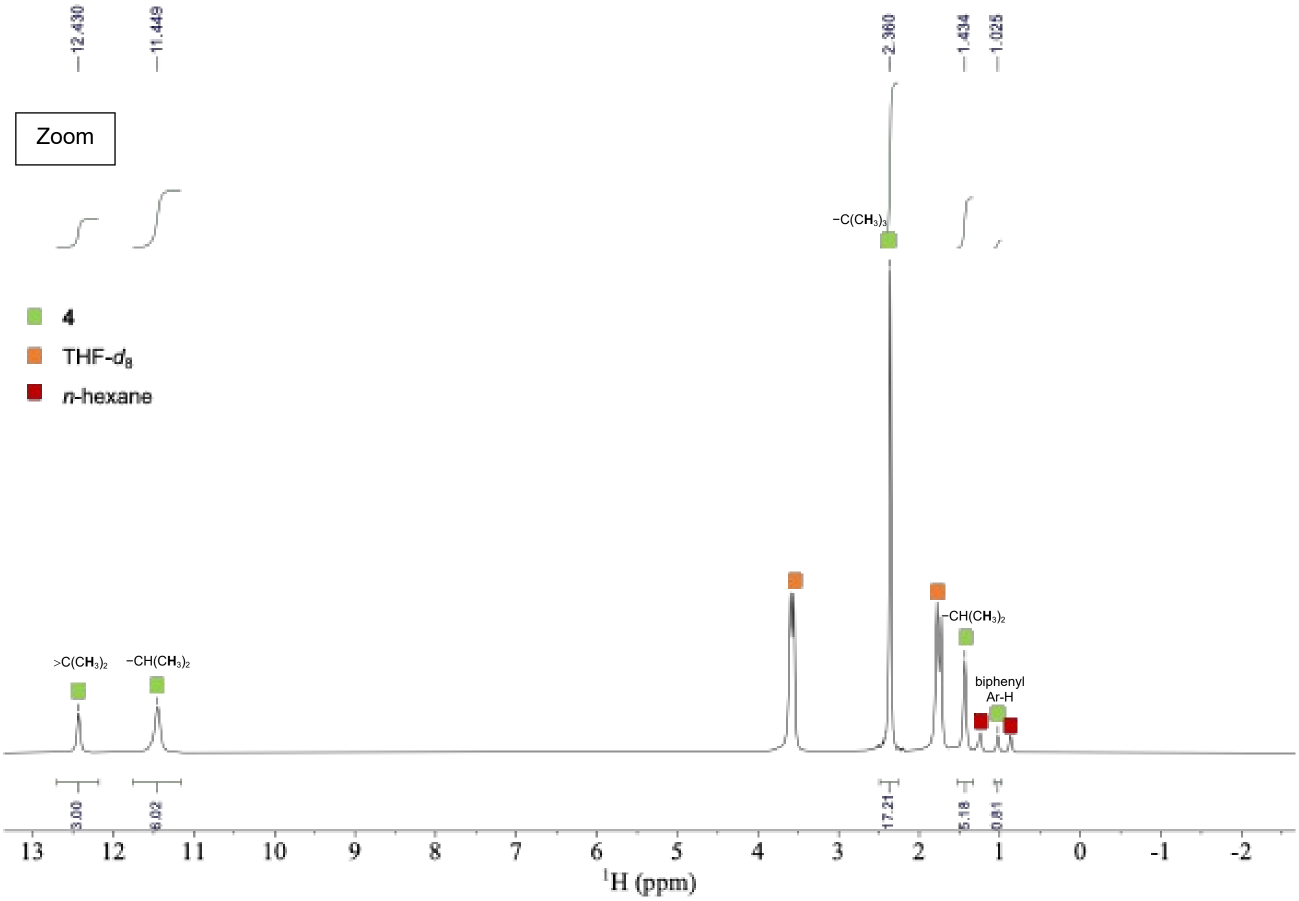

Figure S18. Zoom of the diamagnetic region in **Figure S17**.

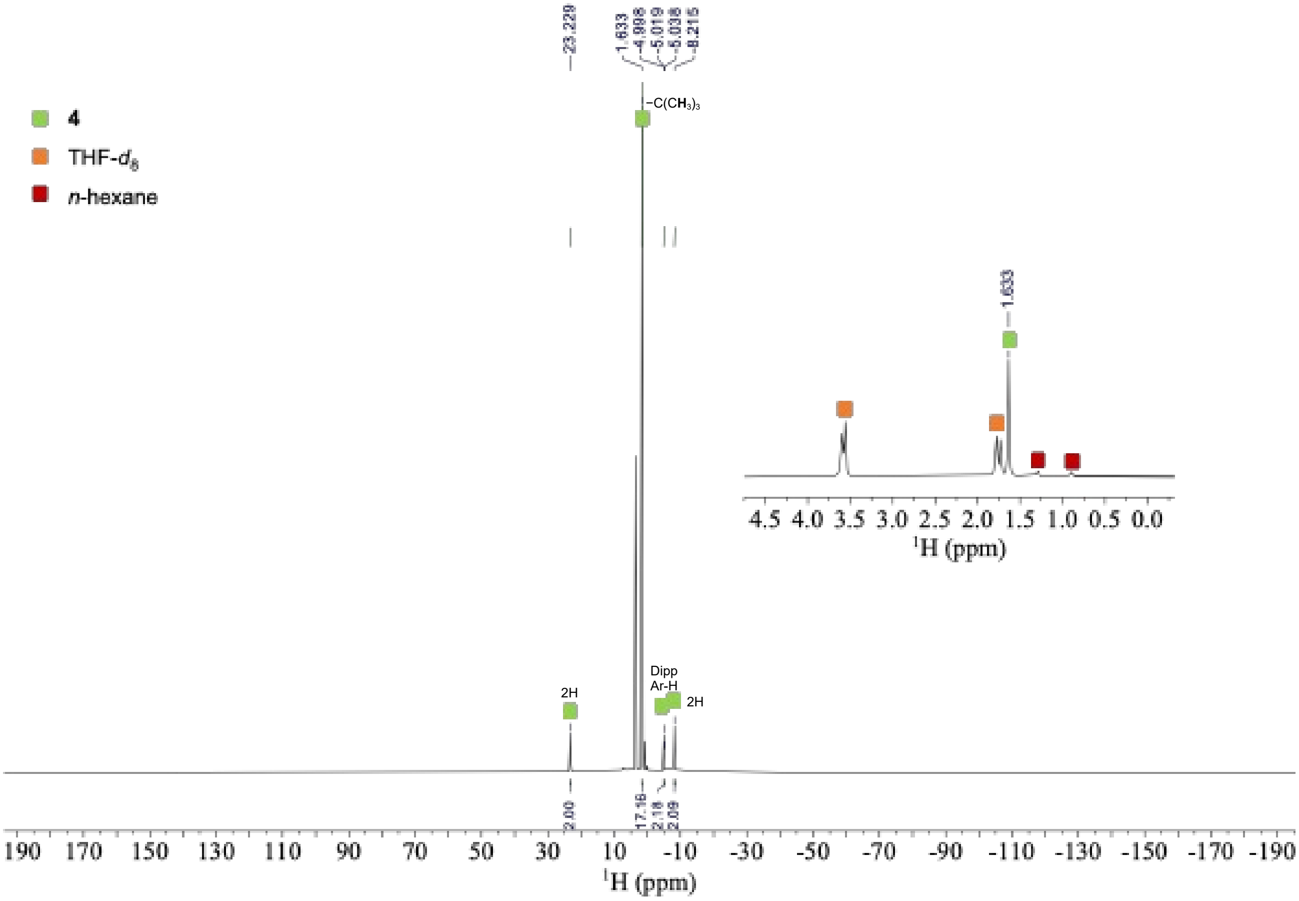

Figure S19. ^{1}H NMR spectrum (400 MHz, THF-d_8, 298 K) of isolated **4**.

S3.5.2. NMR spectra of the reaction mixture of 4 with CO

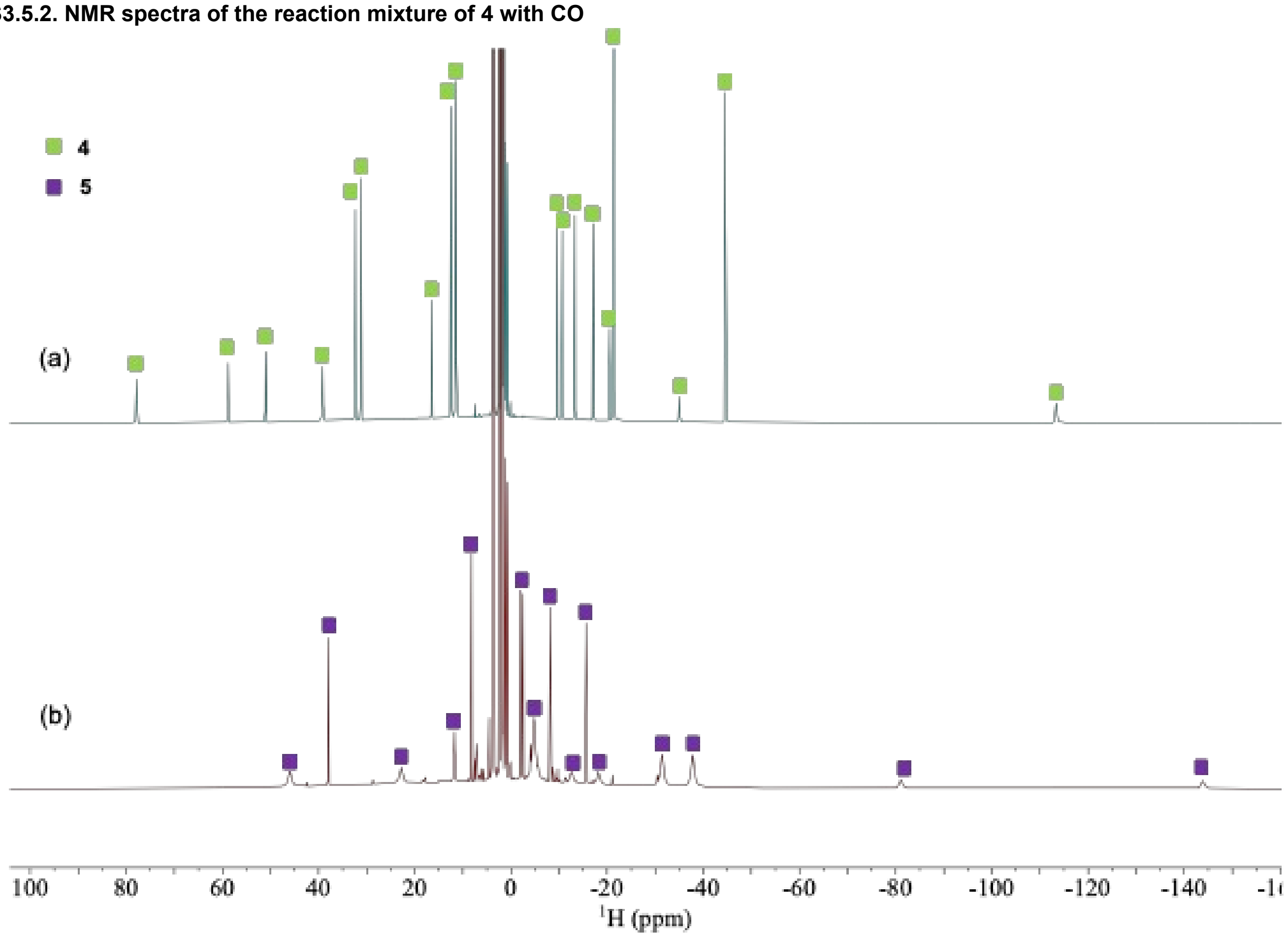

Figure S20. ^{1}H NMR spectrum (400 MHz, THF-d_8, 233 K) of **4** (a) before; (b) immediately after adding CO.

S3.6. NMR spectra of isolated [NNN-U(THF)$_2$(fluorenone)][K(THF)$_4$] (**5**)

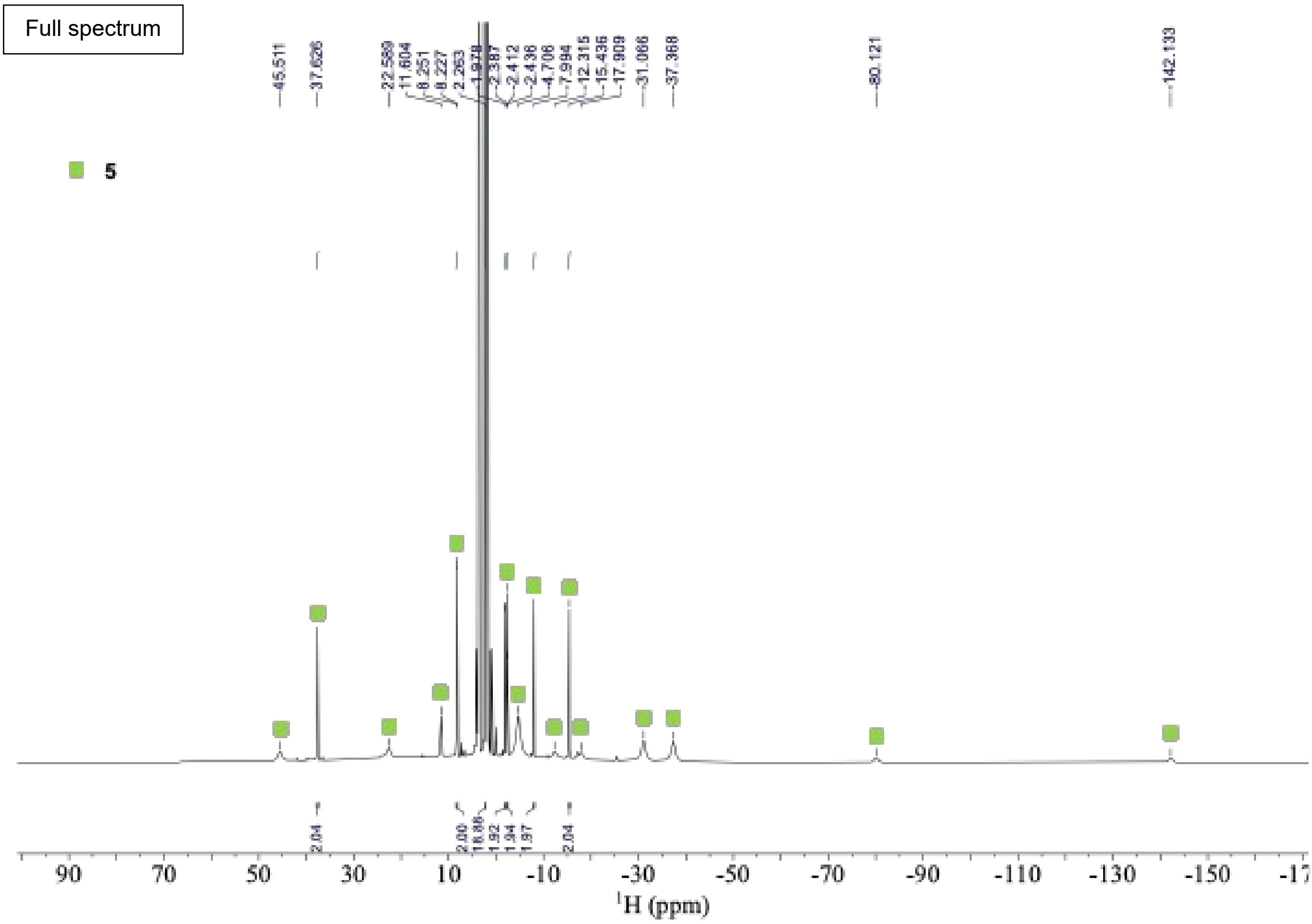

Figure S21. ^{1}H NMR spectrum (400 MHz, THF-d_8, 233 K) of isolated **5**.

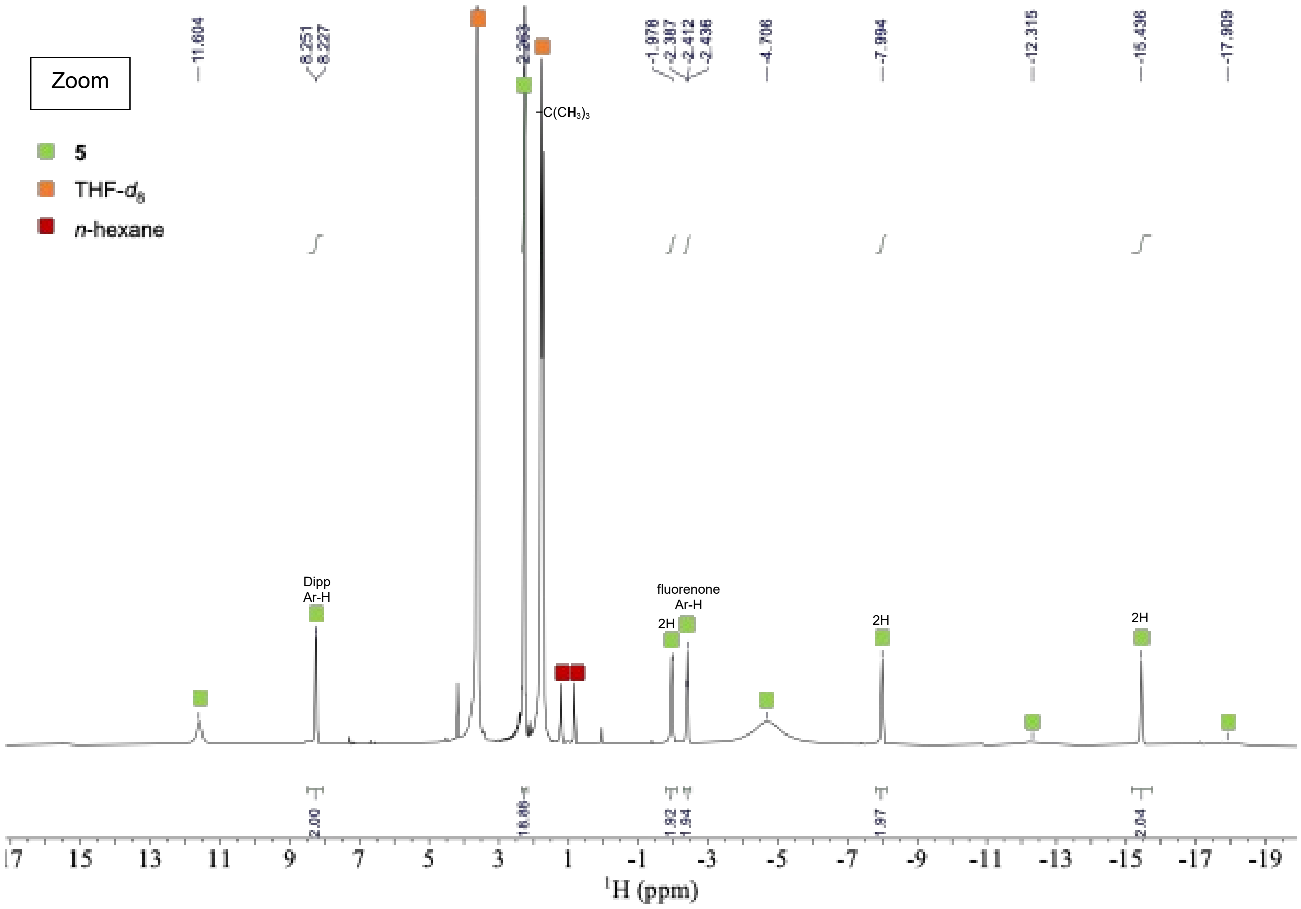

Figure S22. Zoom of the diamagnetic region in **Figure S21**.

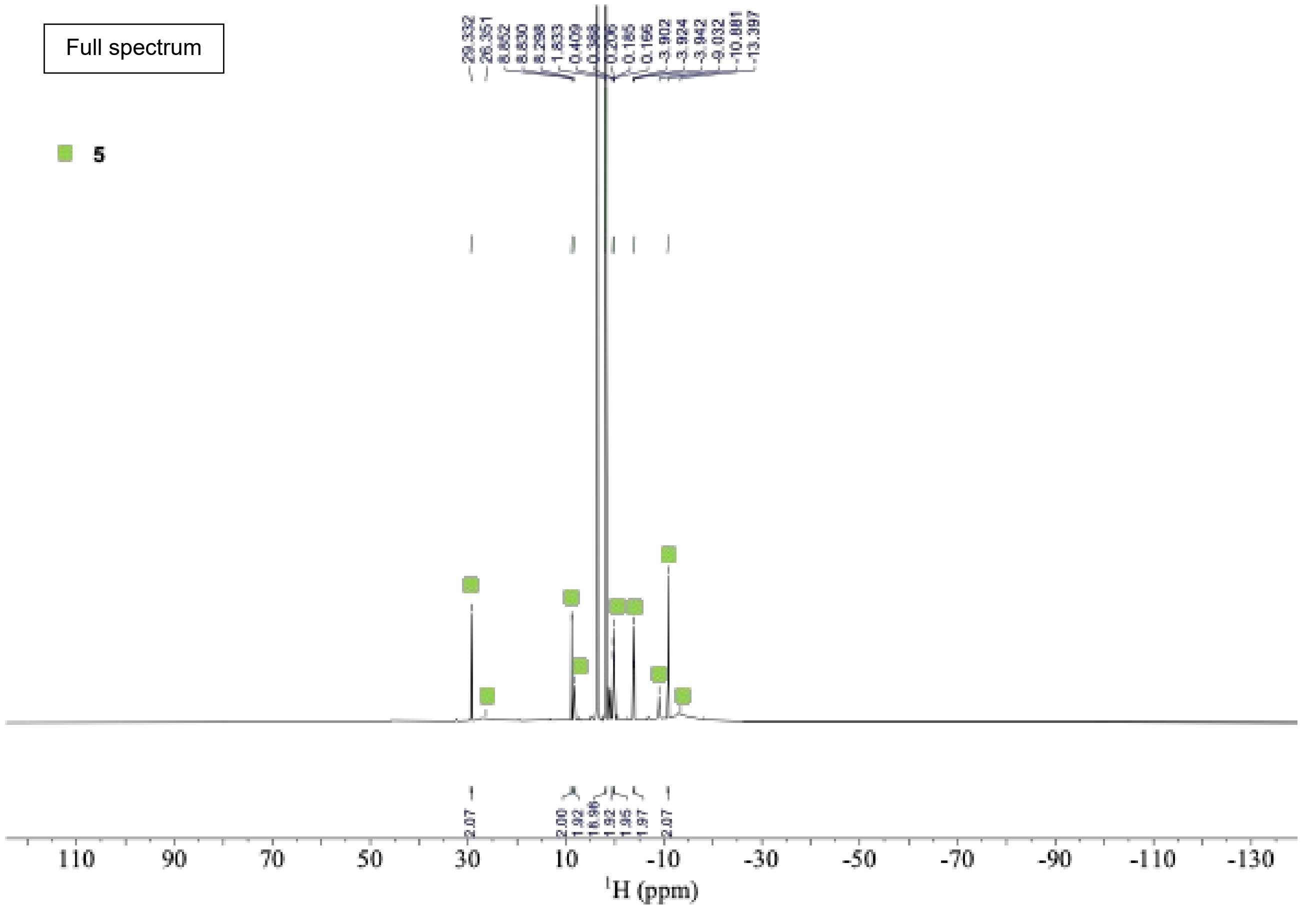

Figure S23. ^{1}H NMR spectrum (400 MHz, THF-d_8, 298 K) of isolated **5**.

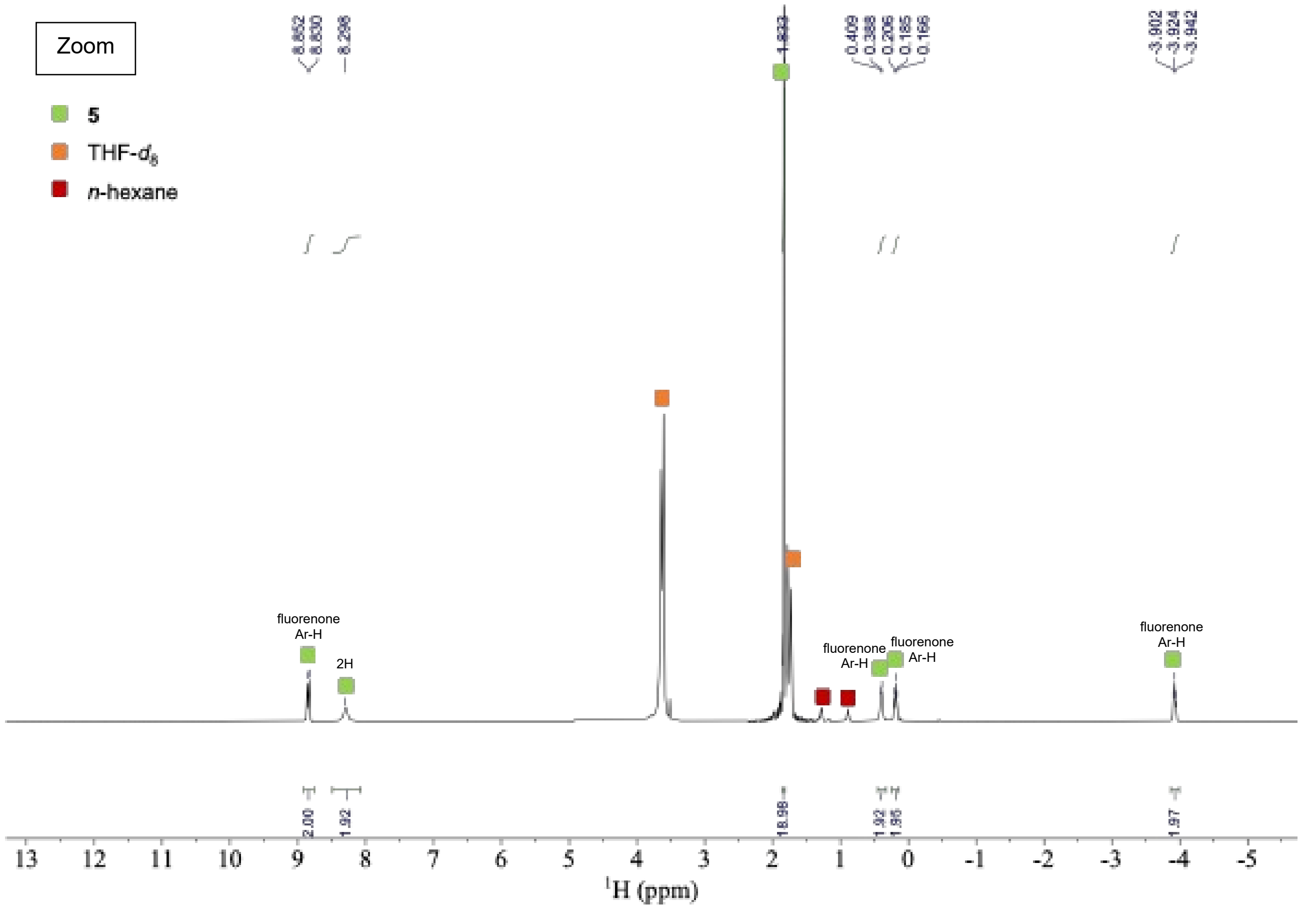

Figure S24. Zoom of the diamagnetic region in **Figure S23**.

S4. X-ray Crystallographic Data

Table S1. Crystal data and structural refinement parameters for compounds **K3NNN**, **1**, **2b** and **2c**.

	**K3NNN**	**1**	**2b**	**2c**
Formula	$C_{119.2}H_{187}K_6N_6O_{6.3}$	$C_{126}H_{188}Cl_4K_2N_6O_8U_2$	$C_{122}H_{186}Cl_3K_3N_6O_7U_2$	$C_{67}H_{102}N_3O_5U$
Color	clear dark yellow	clear dark yellow	lustrous dark black	clear dark brown
Shape	irregular	prism	irregular	prism
Crystal size (mm)	0.39×0.30×0.24	0.19×0.08×0.05	0.30×0.12×0.07	0.19×0.04×0.04
Crystal System	monoclinic	triclinic	monoclinic	monoclinic
Space Group	$P2_1/n$	P-1	Ia	$P2_1/n$
Volume (Å^3)	6049.33(9)	3149.12(10)	12396.0(3)	12895.4(3)
a (Å)	15.91762(13)	11.6846(2)	22.70041(19)	28.4069(4)
b (Å)	14.57188(13)	15.7796(3)	22.5404(3)	15.1709(2)
c (Å)	26.4106(2)	18.5920(3)	26.0053(3)	30.0450(4)
α (°)	90	103.3088(15)	90	90
β (°)	99.0700(8)	94.0723(15)	111.3155(12)	95.1738(13)
γ (°)	90	107.2883(17)	90	90
Z	2	1	4	8
Formula Weight	2039.54	2610.87	2548.47	1267.603
Density (g cm^{-3})	1.120	1.377	1.366	1.306
μ (mm^{-1})	2.321	8.976	9.202	7.454
Temperature (K)	140.00(10)	140.00(10)	140.00(10)	140.00(12)
Total Reflections	42711	26131	103540	67959
Unique Reflections	12452	12208	17248	24945
R_{int}	0.0261	0.0365	0.0383	0.0782
R Indices [I > 2σ(I)]	R_1 = 0.0439	R_1 = 0.0307	R_1 = 0.0478	R_1 = 0.0549
	wR_2 = 0.1244	wR_2 = 0.0723	wR_2 = 0.1233	wR_2 = 0.1399
Largest Diff. Peak and	0.847 and	1.575 and	2.718 and	3.2595 and
Hole (e.A^{-3})	-0.415	-1.818	-2.147	-1.9480
GOF	1.040	1.023	1.017	1.0224
CCDC	2404451	2404452	2442062	2457232

R(int) =∑|Fo2−Fo2(mean)| /∑[Fo2]; I, measured intensities; 'Largest diff. peak and hole', maximum and minimum electron density found in the final Fourier difference map; GOF, goodness of fit (= {∑ [w(Fo2−Fc2)2]/(n−p)}1/2, where n is the number of reflections and p is the total number of parameters refined).

Table S2. Crystal data and structural refinement parameters for complexes **3**, **4** and **5**.

	**3**	**4**	**5**
Formula	$C_{83}H_{118}KN_3O_6U$	$C_{83}H_{122}KN_3O_3U$	$C_{92}H_{134}KN_3O_9U$
Color	metallic dark brown	clear dark brown	clear dark orange
Shape	prism	prism	plate
Crystal size (mm)	0.24×0.20×0.10	0.24×0.10×0.08	0.30×0.26×0.17
Crystal System	monoclinic	monoclinic	triclinic
Space Group	$P2_1/n$	$P2_1/n$	P-1
Volume (Å^3)	7671.68(14)	7821.05(14)	4250.57(15)
a (Å)	15.80343(14)	14.81503(15)	13.3940(3)
b (Å)	22.6493(2)	15.69327(17)	14.0175(2)
c (Å)	21.5753(2)	33.7005(3)	25.0941(5)
α (°)	90	90	89.5052(15)
β (°)	96.5821(9)	93.4484(9)	75.2450(17)
γ (°)	90	90	69.4982(18)
Z	4	4	2
Formula Weight	1530.93	1486.96	1703.14
Density (g cm^{-3})	1.325	1.263	1.331
μ (mm^{-1})	2.223	6.668	6.259
Temperature (K)	139.99(10)	140.00(10)	139.99(10)
Total Reflections	131598	41957	20056
Unique Reflections	36341	15116	20056
R_{int}	0.0560	0.0531	--
R Indices [I > 2σ(I)]	R_1 = 0.0399	R_1 = 0.0402	R_1 = 0.0789
	wR_2 = 0.0747	wR_2 = 0.1008	wR_2 = 0.2238
Largest Diff. Peak and	1.332 and	1.928 and	4.581 and
Hole (e.A^{-3})	-1.371	-2.402	-5.242
GOF	1.023	1.054	1.204
CCDC	2392949	2404453	2404454

R(int) =∑|Fo2−Fo2(mean)| /∑[Fo2]; I, measured intensities; 'Largest diff. peak and hole', maximum and minimum electron density found in the final Fourier difference map; GOF, goodness of fit (= {∑ [w(Fo2−Fc2)2]/(n−p)}1/2, where n is the number of reflections and p is the total number of parameters refined).

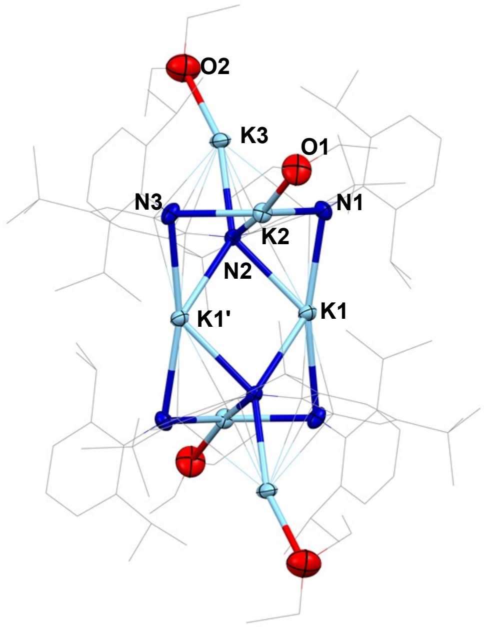

Figure S25. Molecular structure of [NNN-3K(Et_2O)$_2$]$_2$ (**K3NNN**) with thermal ellipsoids drawn at the 50% probability level. Hydrogen atoms and disordered parts of K^+ and Et_2O have been omitted for clarity; the pincer backbones and Et_2O molecules are drawn in wireframe style.

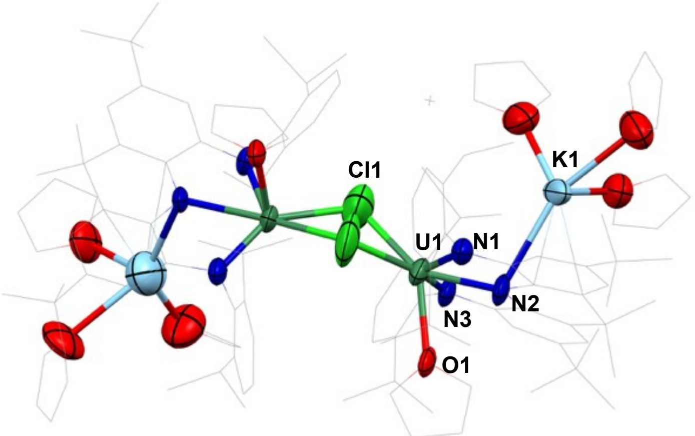

Figure S26. Molecular structure of [NNN-U^{III}Cl(THF)K(THF)$_3$]$_2$ (**2a**) with thermal ellipsoids drawn at the 50% probability level. Hydrogen atoms have been omitted for clarity; the pincer backbones and THF molecules are drawn in wireframe style. The poor quality of the structure allows to determine the connectivity, but prevents discussion of the metrical parameters.

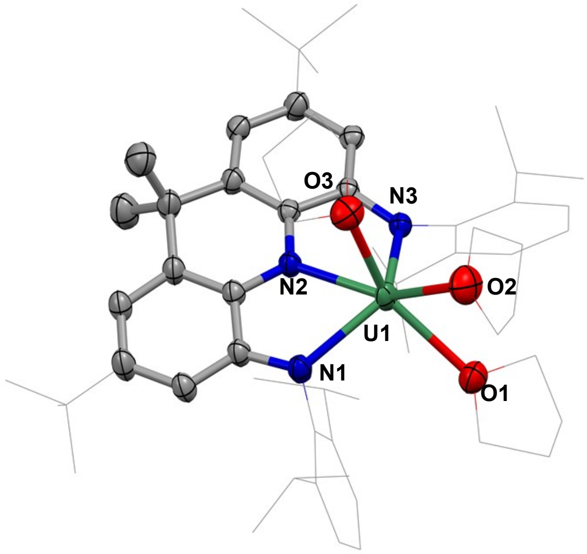

Figure S27. Molecular structure of NNN-$U^{III}(THF)_3$ (**2c**) with thermal ellipsoids drawn at the 50% probability level. Hydrogen atoms, distorted parts and the second molecule in the asymmetric unit of **2c** have been omitted for clarity.

S5. EPR of complexes 2 and 3

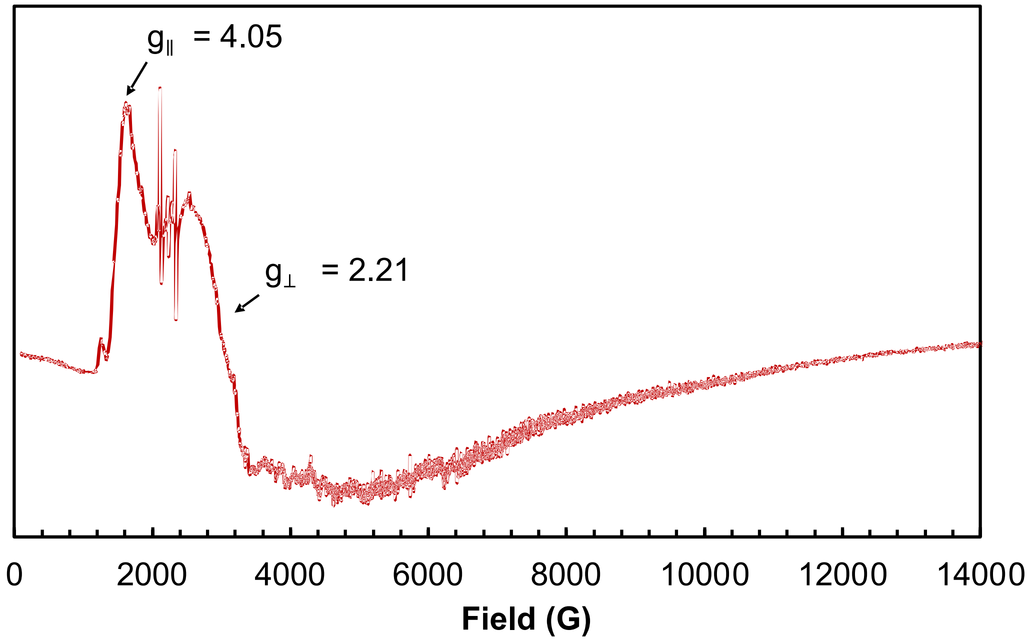

Figure S28. X-band (9.4 GHz) EPR spectrum of **2** in THF (10 mM) at 6 K.

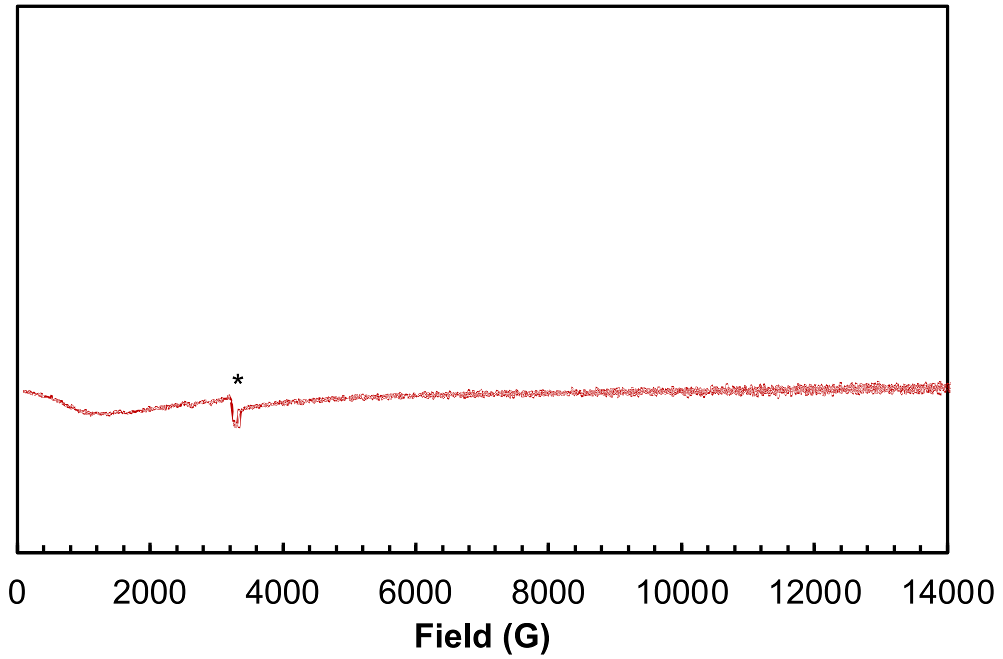

Figure S29. X-band (9.4 GHz) EPR spectrum of complex **3** in THF (10 mM) at 6 K. The * symbol corresponds to the signal coming from the empty cavity.

S6. Computational Details

All the DFT calculations were performed using Gaussian09 suite of programs.[8] Hybrid DFT functional (B3PW91) along with relativistic effective core potentials (RECP) from the Stuttgart-Dresden group in combination with their adapted basis sets for uranium atom and Pople (6-31G**) basis set were used for the rest of the atoms.[9-11] Frequency calculations were performed to locate minima for the optimized structures and for obtaining thermal corrections over the energies.

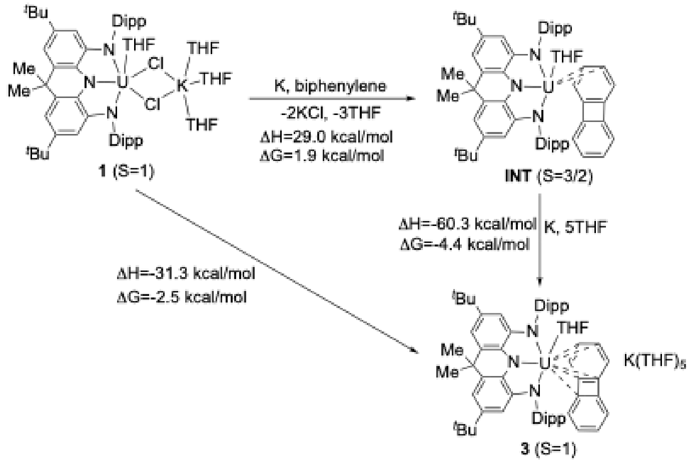

Figure S30. Formation of **3** from **1** via the U(III) intermediate at room temperature.

Table S3. Computed spin density values of uranium/carbon atoms in different complexes/intermediates/transition states.

**1**, U=2.18	**INT**, U=2.58, C=0.18, 0.14,0.18
**3,** U=2.27	**3-noK**, U=2.18
**TS1**, U= 2.45, C=-0.10,-0.08	**4-noK**, U=2.18
**4-THF**, U=2.17	**TS2**, U=2.17
**5-noK**, U=2.25	

All attempts to converge another spin state failed since the system was willing to localize only 2 unpaired electrons at the uranium center for all complexes.

Table S4 Comparison of selected structural parameters (angstrom and degrees) between computed and experimental structures for **3.**

Atom label	x-ray	DFT
U1-O2	2.48	2.52
U1-N3	2.39	2.44
U1-N4	2.40	2.38
U1-N5	2.34	2.31
U1-C27	2.63	2.69
U1-C47	2.80	2.82
U1-C69	2.81	2.76
U1-C91	2.66	2.63
U1-C94	2.65	2.63
U1-C126	2.65	2.66
N5-U1-O2	76.6	80.2
N5-U1-X (centroid of phenyl ring)	179.5	169.7

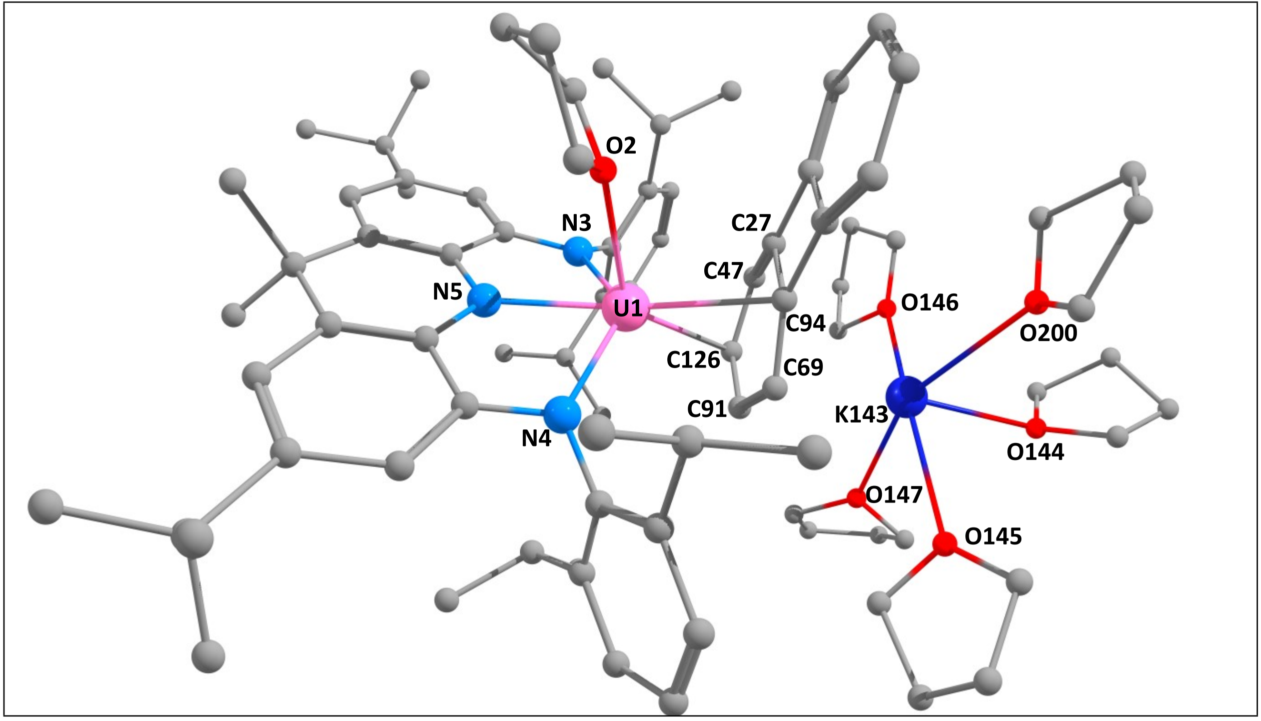

Table S5. Comparison of selected structural parameters (angstrom and degrees) between computed (**4-noK**) and experimental (**4**) structures.

Atom label	x-ray	DFT
U1-N2	2.33	2.33
U1-N3	2.32	2.32
U1-N4	2.35	2.29
U1-C23	2.52	2.48
U1-C42	2.49	2.42
N4-U1-C23	147.9	164.4
C23-U1-C42	68.6	70.7

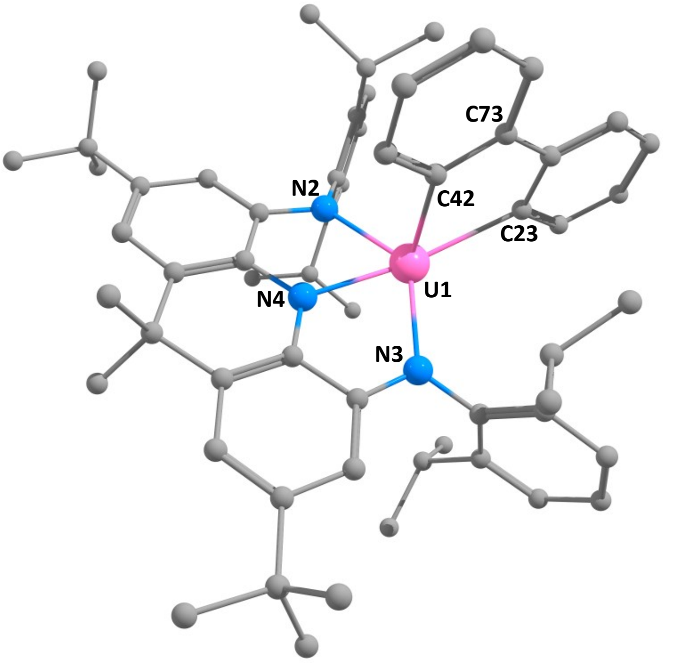

Table S6. Comparison of selected structural parameters (angstrom and degrees) between computed (**5-noK**) and experimental (**5**) structures.

Atom label	x-ray	DFT
U1-N2	2.34	2.29
U1-N4	2.35	2.36
U1-N5	2.39	2.41
U1-O148	2.18	2.17
C147-O148	1.38	1.36
U1-O3	2.57	2.64
N2-U1-O148	152.0	147.7
U1-O148-C147	90.4	91.7

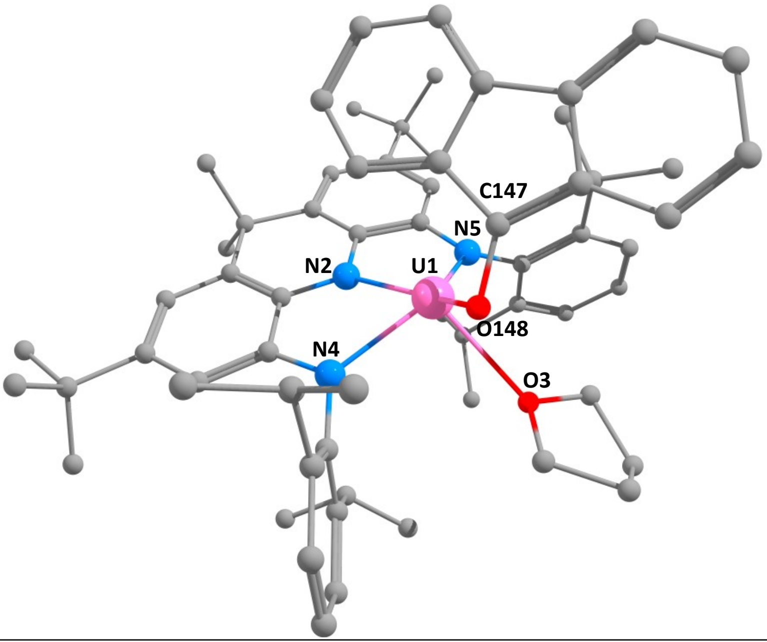

S7. References

[1] (a) I. A. Khan, H. S. Ahuja, K. W. Bagnall, L. Sinf, in *Inorganic Syntheses*, 1982, pp. 187-190; (b) J. L. Kiplinger, D. E. Morris, B. L. Scott, C. J. Burns, *Organometallics* 2002, ***21***, 5978-5982.

[2] D. E. Bergbreiter, J. M. Killough, *J. Am. Chem. Soc.* 1978, ***100***, 2126-2134.

[3] M. K. Mondal, L. Zhang, Z. Feng, S. Tang, R. Feng, Y. Zhao, G. Tan, H. Ruan, X. Wang, *Angew. Chem., Int. Ed.* 2019, ***58***, 15829-15833.

[4] CrysAlisPro (Rigaku, V1.171.43.107a, 2024).

[5] G. M. Sheldrick, *Acta Cryst. Section A* 2015, ***71,*** 3-8.

[6] O. V. Dolomanov, L. J. Bourhis, R. J. Gildea, J. A. K. Howard, H. Puschmann, *J. App. Crystallogr.* 2009, ***42***, 339-341.

[7] G. M. Sheldrick, *Acta Crystallogr. Sect. C* 2015, ***71***, 3-8.

[8] Gaussian 09, Revision D.01: M. J. Frisch, G. W. Trucks, H. B. Schlegel, G. E. Scuseria, M. A. Robb, J. R. Cheesman, G. Scalmani, V. Barone, B. Mennucci, G. A. Petersson, H. Nakatsuji, M. Caricato, X. Li, H. P. Hratchian, A. F. Izmaylov, J. Bloino, G. Zheng, J. L. Sonnenberg, M. Hada, M. Ehara, K. Toyota, R. Fukuda, J. Hasegawa, M. Ishida, T. Nakajima, Y. Honda, O. Kitao, H. Nakai, T. Vreven, J. A., Jr. Montgomery, J. E. Peralta, F. Ogliaro, M. Bearpark, J. J. Heyd, E. Brothers, K. N. Kudin, V. N. Staroverov, R. Kobayashi, J. Normand, K. Raghavachari, J. C. Burant, S. S. Iyengar, J. Tomasi, M. Cossi, N. Rega, M. J. Millam, M. Klene, J. E. Knox, J. B. Cross, V. Bakken, C. Adamo, J. Jaramillo, R. Gomperts, R. E. Stratmann, O. Yazyev, A. J. Austin, R. Cammi, C. Pomelli, J. W. Ochterski, R. L. Martin, K. Morokuma, V. G. Zakrzewski, G. A. Voth, P. Salvador, J. J. Dannenberg, S. Dapprich, A. D. Daniels, O. Farkas, J. B. Foresman, J. V. Ortiz, J. Cioslowski and D. J. Fox, Gaussian Inc., 2009, Wallingford CT.

[9] (a) A. D. Becke, *J. Chem. Phys*. 1993, ***98***, 5648; (b) K. Burke, J. P. Perdew, W. Yang, in Electronic Density Functional Theory: Recent Progress and New Directions, Eds: J. F. Dobson, G. Vignale, M. P. Das, Plenum, New York, 1998

[10] A. Moritz, X. Cao and M. Dolg, *Theor. Chem. Acc*. 2007, ***118***, 845

[11] (a) P. C. Hariharan and J. A. Pople, *Theor. Chim. Acta* 1973, **28**, 213; (b) W. J. Hehre, R. Ditchfield and J. A. Pople, *J. Chem. Phys*. 1972, ***56***, 2257.
